# Effects of 4‑Alkoxy/Amino-7-Chloroquinolines on *Aedes aegypti* and *Artemia salina* Larvae: The Search for Safer Larvicides to Combat Epidemiological Dengue in the Americas

**DOI:** 10.1021/acsomega.5c05060

**Published:** 2025-08-15

**Authors:** Everton P. Silva, Agenor P. Luz-Filho, Abraão P. Sousa, Edilson B. Alencar-Filho, Vanessa C. Santos, Luana B. R. Silva, Priscila S. V. Lima, Helivaldo D. S. Souza, Petrônio F. Athayde-Filho, Gabriela F. Fiss

**Affiliations:** † Department of Chemistry, Universidade Federal da Paraíba (UFPB), João Pessoa 58051-900, Brazil; ‡ Department of Pharmaceutical Sciences, 74373 Universidade Federal do Vale do São Francisco (UNIVASF), Petrolina 56304-917, Brazil; § Department of Chemistry, 28118 Universidade Federal de Santa Maria (UFSM), Santa Maria 97105-900, Brazil

## Abstract

*Aedes aegypti*, the main transmitter of arboviruses responsible for several arboviruses, such as dengue, is highly prevalent in Latin America, especially in Brazil. Controlling this vector is urgent for public health, since it is more advantageous to prevent than to treat the diseases it causes. The use of chemical control methods, such as larvicides, is strategic. In this sense, the search for environmentally safer larvicides has driven the development of compounds with activity against *Ae. aegypti* and low toxicity to nontarget organisms. In this study, a rational design of ten 4-alkoxy/amino-7-chloroquinolines was carried out to investigate their larvicidal and toxic properties, from prediction to *in vitro* assays. Larvicidal tests revealed seven bioactives against *Ae. aegypti* (LC_50_ ≤ 69.18 ppm), of which compound **3** was very active (LC_50_ = 2.51 ppm) and compound **6′** was very highly active (LC_50_ = 0.02 ppm), surpassing the standard larvicide Spinosad (LC_50_ = 0.05 ppm). Structural analysis indicated that the −O–pentyl and −O–ethyl–Cl moieties at the 4-position of 7-chloroquinolines improved larvicidal activity when compared to 4,7-dichloroquinoline. Regarding the *in vitro* ecotoxicity study, bioactives **1**, **6′**, **7′**, and **9′** showed moderate toxicity on *Artemia salina* larvae (LC_50_ = 101.68–314.58 μg·mL^–1^). The best results demonstrated that compound **6′**, potentially nonmutagenic, nonhepatotoxic, and predicted not to cause skin sensitization, was 15 729 times more toxic to *Ae. aegypti* than to the nontarget organism *A. salina*, emerging as a safer larvicide.

## Graphical abstract

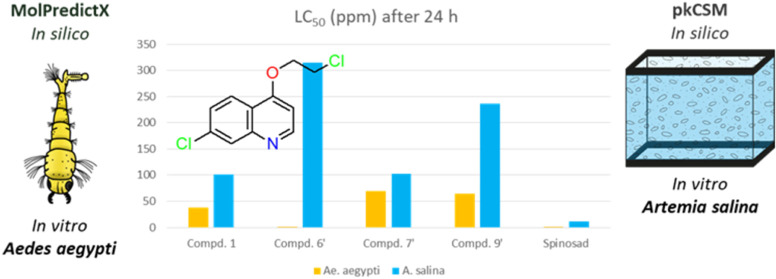

## Introduction

The infected female *Aedes aegypti* mosquito is recognized as a vector of arbovirus transmission, responsible for arboviruses such as chikungunya, dengue, yellow fever, and Zika.^1^ According to the Pan American Health Organization (PAHO),^2^ although *Ae. aegypti* is present in almost all countries in the Western Hemisphere, the highest incidence occurs in Latin America, especially in Brazil, with 10 267 077 cases of dengue in 2024 alone. Among the countries in the region, only Canada remains free of the arbovirus.

According to the Epidemiological Report of 2024,^2^ 13 064 226 suspected cases of dengue fever in the Americas were reported. Of these, 22 807 were classified as severe dengue (0.17%), and 8348 were classified as deaths, yielding a case fatality rate (CFR) of 0.054%. This represents an increase of 284% compared to 2023, and 271% to the average of the last 5 years.

*Ae. aegypti* goes through four stages before reaching the mosquito form: egg, larva, pupa, and adult form. Under favorable environmental conditions, an egg takes between eight and 12 days to become a mosquito. Female *Ae. aegypti* mosquitoes, mainly active during the day, need human blood for the development of their eggs and metabolism.^3,4^ Controlling their populations is considered a public health issue, and there is scientific evidence that controlling *Ae. aegypti* costs governments less than treating diseases caused by this vector.^5^

Vector control methods can be mechanical, biological, genetic, chemical, or by monitoring. Chemical control is based on the use of insecticides, which attack the adult form of the insect, or larvicides, which combat the larvae of the vector. In this sense, 4,7-dichloroquinoline (Figure 1) was studied against dengue vectors, which showed significant larvicidal and pupicidal properties against *Anopheles stephensi* and *Ae. aegypti*, with 50% lethal concentration (LC_50_) ranging from 4.40 (larvae) to 7.95 (pupae) ppm and 5.01 (larvae) to 10.66 (pupae) ppm after 24 h, respectively.^6^

**Figure 1:**
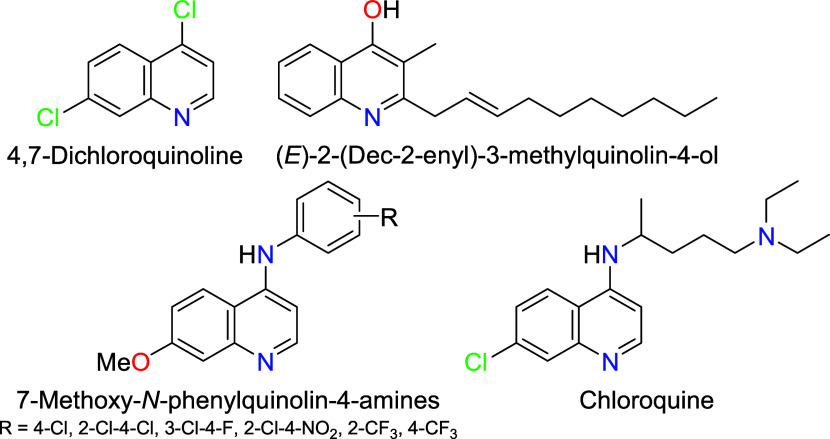
Promising quinoline derivatives.

Additionally, (*E*)-2-(dec-2-enyl)-3-methylquinolin-4-ol (Figure 1), isolated in petroleum ether extracts from 72 h culture supernatant of *Pseudomonas aeruginosa* KUN2, showed 100% mortality on the fourth instar larvae of *Ae. aegypti* after 24 h.^7^ In another study,^8^ quinoline derivatives exhibited a significant activity on the third instar larvae of *Ae. aegypti* after 24 h, with the best result for an LC_50_ value equal to 90.58 μg·mL^–1^. Alternatively, 7-methoxy-*N*-phenylquinolin-4-amines (Figure 1) have been investigated as dengue protease inhibitors.^9,10^

As for Chloroquine (Figure 1), a privileged antimalarial 4-substituted 7-chloroquinoline,^11,12^ the chlorine substituent has been shown to be an essential structural feature in marketed drugs, which has led to it being known as “magic chloro”.^13^ As a result, 4-substituted 7-chloroquinolines have been studied supramolecularly^14^ due to their broad spectrum bioactivities.

As important as the design of active compounds is the delivery of more selective and safer bioactives, *i.e.*, those that are less toxic to human organisms and the environment. The scientific community has reported interest in both cytotoxicity and ecotoxicity.^15^ Nguyen et al.^16^ related that the 2-methyl-3,4-dihydroquinazolin-4-one moiety exhibited activity against *Ae. aegypti* larvae, with LC_50_ values of 2.08–4.20 μg·mL^–1^ after 72 h, which did not exhibit ecotoxicity on *Diplonychus rusticus* at 25 μg·mL^–1^.

On the potential disposal of drugs in the aquatic environment, *in vitro* toxicity on *Artemia salina* larvae has been used to evaluate the ecotoxicity of bioactives, which may discriminate cytotoxic compounds.^15^ Our research group has been developing more selective and cleaner 4-substituted quinolines.^17–19^ Now, considering the promising larvicidal activity of quinoline derivatives^6–8^ as well as the concern about the impact on the environment, we rationally designed ten 4-alkoxy/amino-7-choroquinolines to study their effects on *Ae. aegypti* and *A. salina* larvae, focusing on how oxygen or nitrogen at the 4-position and variations in side chain length with and without a terminal chlorine atom affect larvicidal activity.

## Materials and Methods

### Materials

All common reagents were purchased from commercial suppliers and used without further purification. Compounds were synthesized at Laboratório de Bioenergia e Síntese Orgânica (LPBS-UFPB), and melting points were measured using QUIMIS equipment, model Q340S23 (Diadema, Brazil). ^1^H and ^13^C Nuclear Magnetic Resonance (NMR) spectra were acquired at Laboratório Multiusuário de Caracterização e Análise (LMCA-UFPB), on Bruker Ascend (Coventry, United Kingdom) and Bruker (Billerica, Massachusetts) spectrometers at 400 and 500 MHz for ^1^H, respectively, using 5 mm tubes at 298 K. Infrared (IR) spectra were acquired at Laboratório de Síntese Orgânica Medicinal (LASOM-UFPB), on a Shimadzu spectrometer, model IRPrestige-21 (Kyoto, Japan), using KBr pellets. High-resolution mass spectrometry (HRMS) analyses were acquired at LMCA-UFPB, on a Shimadzu HPLC (Kyoto, Japan) coupled to a Bruker MicrOTOF II (Billerica, Massachusetts), with an electrospray ion (ESI) source and reported as *m*/*z* (relative intensity) for molecular ion [M + H]^+^. Acquisition Parameters: Ion Polarity Positive, Capillary 4500 V, End Plate Offset -500 V, Nebulizer 4.0 bar, Dry Heater 200 °C, Dry Gas 8.0 L·min^–1^, Divert Valve Waste.

### Experimental Procedures for the Synthesis of 4-Alkoxy/amino-7-choroquinolines (**1–10**)

According to methods previously described by Fiss et al.,^19^ compounds **1–10** were obtained in yields ranging from 81 to 99%. Our research group previously described full data for compounds **2–10**.^19^

### 7-Chloro-4-ethoxyquinoline (**1**)

Yield: 95% (9.5 mmol, 1.97 g); white solid; m.p.: 105–106 °C (99–101 °C);^20^ ^1^H NMR (500 MHz, CDCl_3_): δ = 8.70 (d, *J* = 5.2 Hz, 1H, H_Ar_), 8.14 (d, *J* = 8.9 Hz, 1H, H_Ar_), 8.00 (d, *J* = 2.0 Hz, 1H, H_Ar_), 7.42 (dd, *J* = 8.9, 2.1 Hz, 1H, H_Ar_), 6.68 (d, *J* = 5.3 Hz, 1H, H_Ar_), 4.24 (q, *J* = 7.0 Hz, 2H, CH_2_), 1.56 (t, *J* = 7.0 Hz, 3H, CH_3_) ppm; ^13^C NMR (126 MHz, CDCl_3_): δ = 161.7, 152.6, 149.8, 135.7, 127.9, 126.5, 123.6, 120.0, 101.0 (9 × C_Ar_), 64.4 (CH_2_), 14.5 (CH_3_) ppm; IR (KBr): ν = 3062, 3039 (H_Ar_), 2985, 2958, 2943 (H_alkanic_), 1612 (C=N), 1577, 1500, 1469, 1450 (C=C), 1311, 1280, 1234, 1195, 1157, 1122, 1068 (C–O), 871, 833, 813, 763, 748 (Ar), 644, 624 (C–Cl) cm^–1^.

### General Procedure for the Synthesis of 4-Alkoxy/amino-7-choroquinolines (**6′–10′**)

Compounds **6′–10′** were synthesized according to a method previously reported by Oliveira et al.,^21^ with some adjustments. A mixture of alcohol (**6–10**, 3 mmol), thionyl chloride (SOCl_2_, 30 mmol), and dichloromethane (DCM, 3 mL) was maintained under magnetic stirring at 25 °C for 6 h, which was monitored by thin layer chromatography in ethyl acetate (AcOEt). Afterward, the reactional mixture was evaporated under reduced pressure. Then, the reactional residue was washed with saturated sodium bicarbonate solution (60 mL), and the precipitate was filtered with excess water under reduced pressure. Compounds **6′–10′** were obtained in yields ranging from 76 to 99%. Full NMR, IR, and HRMS spectra for compounds **6′–8′** are available in Figures S1–S12.

### 7-Chloro-4-(2-chloroethoxy)­quinoline (**6′**)

Yield: 86% (2.58 mmol, 0.62 g); *R*_f_: 0.4 (AcOEt); white solid; m.p.: 112–114 °C; ^1^H NMR (500 MHz, CDCl_3_): δ = 8.73 (d, *J* = 5.2 Hz, 1H, H_Ar_), 8.16 (d, *J* = 8.9 Hz, 1H, H_Ar_), 8.02 (d, *J* = 2.1 Hz, 1H, H_Ar_), 7.46 (dd, *J* = 8.9, 2.1 Hz, 1H, H_Ar_), 6.69 (d, *J* = 5.3 Hz, 1H, H_Ar_), 4.44 (t, *J* = 5.7 Hz, 2H, CH_2_), 3.96 (t, *J* = 5.6 Hz, 2H, CH_2_) ppm; ^13^C NMR (126 MHz, CDCl_3_): δ = 160.9, 152.4, 149.9, 136.1, 128.0, 126.9, 123.5, 119.7, 101.0 (9 × C_Ar_), 68.3 (CH_2_), 41.4 (CH_2_) ppm; IR (KBr): ν = 3074, 3039, 3024 (H_Ar_), 2974, 2935, 2877, 2850, 2823 (H_alkanic_), 1612 (C=N), 1566, 1500, 1458 (C=C), 1300, 1276, 1234, 1192, 1157, 1118, 1083, 1068 (C–O), 864, 810, 767, 744 (Ar), 671, 640 (C–Cl) cm^–1^; HRMS (ESI): calcd for C_11_H_10_Cl_2_NO ([M + H]^+^) 242.0134, found 242.0139.

### 7-Chloro-4-(3-chloropropoxy)­quinoline (**7′**)

Yield: 88% (2.64 mmol, 0.67 g); *R*_f_: 0.4 (AcOEt); white solid; m.p.: 84–86 °C; ^1^H NMR (500 MHz, CDCl_3_): δ = 8.73 (d, *J* = 5.2 Hz, 1H, H_Ar_), 8.10 (d, *J* = 8.9 Hz, 1H, H_Ar_), 8.02 (d, *J* = 2.1 Hz, 1H, H_Ar_), 7.44 (dd, *J* = 8.9, 2.1 Hz, 1H, H_Ar_), 6.74 (d, *J* = 5.2 Hz, 1H, H_Ar_), 4.35 (t, *J* = 5.9 Hz, 2H, CH_2_), 3.82 (t, *J* = 6.3 Hz, 2H, CH_2_), 2.40 (p, *J* = 6.0 Hz, 2H, CH_2_) ppm; ^13^C NMR (126 MHz, CDCl_3_): δ = 161.3, 152.6, 149.8, 135.9, 128.0, 126.7, 123.3, 119.8, 101.1 (9 × C_Ar_), 65.1 (CH_2_), 41.2 (CH_2_), 31.9 (CH_2_) ppm; IR (KBr): ν = 3101, 3043 (H_Ar_), 2966, 2931, 2889 (H_alkanic_), 1612 (C=N), 1577, 1570, 1500, 1469, 1450 (C=C), 1311, 1276, 1246, 1215, 1195, 1180, 1157, 1118, 1076 (C–O), 840, 817, 798, 767, 748 (Ar), 648, 624 (C–Cl) cm^–1^; HRMS (ESI): calcd for C_12_H_12_Cl_2_NO ([M + H]^+^) 256.0290, found 256.0294.

### 7-Chloro-4-(4-chlorobutoxy)­quinoline (**8′**)

Yield: 76% (2.28 mmol, 0.61 g); *R*_f_: 0.43 (AcOEt); white solid; m.p.: 89–91 °C; ^1^H NMR (500 MHz, CDCl_3_): δ = 8.71 (d, *J* = 5.1 Hz, 1H, H_Ar_), 8.11 (d, *J* = 8.7 Hz, 1H, H_Ar_), 8.01 (d, *J* = 2.1 Hz, 1H, H_Ar_), 7.43 (dd, *J* = 8.9, 2.1 Hz, 1H, H_Ar_), 6.69 (d, *J* = 5.4 Hz, 1H, H_Ar_), 4.22 (t, *J* = 5.9 Hz, 2H, CH_2_), 3.66 (t, *J* = 6.2 Hz, 2H, CH_2_), 2.15–2.02 (m, 4H, 2 × CH_2_) ppm; ^13^C NMR (126 MHz, CDCl_3_): δ = 161.5, 152.6, 149.8, 135.8, 128.0, 126.6, 123.4, 119.9, 101.0 (9 × C_Ar_), 67.8 (CH_2_), 44.6 (CH_2_), 29.3 (CH_2_), 26.4 (CH_2_) ppm; IR (KBr): ν = 3035 (H_Ar_), 2989, 2954, 2866 (H_alkanic_), 1612 (C=N), 1566, 1500, 1469 (C=C), 1311, 1280, 1246, 1195, 1153, 1114, 1072, 1033 (C–O), 844, 817, 798, 771, 748, 725 (Ar), 644, 624 (C–Cl) cm^–1^; HRMS (ESI): calcd for C_13_H_14_Cl_2_NO ([M + H]^+^) 270.0447, found 270.0442.

### 7-Chloro-*N*-(2-chloroethyl)­quinolin-4-amine (**9′**)

Yield: 99% (2.97 mmol, 0.71 g); white solid; m.p.: 159–160 °C (154–155 °C);^22^ ^1^H NMR (400 MHz, CDCl_3_): δ = 8.55 (d, *J* = 5.3 Hz, 1H, H_Ar_), 7.97 (d, *J* = 2.1 Hz, 1H, H_Ar_), 7.73 (d, *J* = 9.0 Hz, 1H, H_Ar_), 7.37 (dd, *J* = 8.9, 2.1 Hz, 1H, H_Ar_), 6.42 (d, *J* = 5.4 Hz, 1H, H_Ar_), 5.62 (s, 1H, NH), 3.83 (t, *J* = 5.8 Hz, 2H, CH_2_), 3.70 (q, *J* = 5.7 Hz, 2H, CH_2_) ppm; ^13^C NMR (101 MHz, CDCl_3_): δ = 152.0, 149.2, 149.2, 135.2, 128.8, 125.8, 121.1, 117.4, 99.3 (9 × C_Ar_), 44.5 (CH_2_), 42.5 (CH_2_) ppm; IR (KBr): ν = 3201 (H–N), 3105, 3059 (H_Ar_), 3001, 2962 (H_alkanic_), 1608 (C=N), 1577, 1543, 1489 (C=C), 1280, 1249, 1203, 1180, 1161, 1138, 1080 (C–N), 875, 848, 837, 810, 767, 752, 702 (Ar), 663, 640, 621 (C–Cl) cm^–1^.

### 7-Chloro-*N*-(3-chloropropyl)­quinolin-4-amine (**10′**)

Yield: 81% (2.43 mmol, 0.61 g); white solid; m.p.: 159–160 °C (154–155 °C);^22^ ^1^H NMR (400 MHz, DMSO-*d*_6_): δ = 8.50–8.45 (m, 3H, NH, 2 × H_Ar_), 7.91 (d, *J* = 2.2 Hz, 1H, H_Ar_), 7.57 (dd, *J* = 9.0, 2.2 Hz, 1H, H_Ar_), 6.67 (d, *J* = 6.3 Hz, 1H, H_Ar_), 3.78 (t, *J* = 6.5 Hz, 2H, CH_2_), 3.52 (q, *J* = 6.6 Hz, 2H, CH_2_), 2.13 (p, *J* = 6.6 Hz, 2H, CH_2_) ppm; ^13^C NMR (101 MHz, DMSO-*d*_6_): δ = 152.7, 147.4, 143.8, 135.6, 125.4, 125.0, 123.2, 116.5, 98.6 (9 × C_Ar_), 43.0 (CH_2_), 40.1 (CH_2_), 30.7 (CH_2_) ppm; IR (KBr): ν = 3217 (H–N), 3089, 3020 (H_Ar_), 2954, 2870, 2835 (H_alkanic_), 1612 (C=N), 1589, 1550, 1489, 1450 (C=C), 1300, 1280, 1253, 1238, 1211, 1165, 1138, 1118, 1080 (C–N), 879, 852, 821, 802, 763, 729 (Ar), 648 (C–Cl) cm^–1^.

### *In Silico*/*In Vitro* Larvicidal Activity on *Ae. aegypti*

*In silico* larvicidal activity for 4-alkoxy/amino-7-choroquinolines (**1–5** and **6′–10′**), 4,7-dichloroquinoline, and Spinosad on *Ae. aegypti* was predicted through the open-access electronic site MolPredictX^23^ (https://www.molpredictx.ufpb.br). According to a method previously reported by Alencar-Filho et al.,^24^ *in vitro* larvicidal activity of 4-alkoxy/amino-7-choroquinolines (**1–5** and **6′–10′**) and 4,7-dichloroquinoline was evaluated on second and third instar larvae (L2–L3) of *Ae. aegypti*. *In vitro* assays on *Ae. aegypti* larvae were performed at Laboratório de Modelagem Molecular Aplicada à Farmácia (LAMMAF-UNIVASF). *Ae. aegypti* larvae were provided by Moscamed Brasil (Juazeiro, Brazil), whose eggs hatched into larvae according to a protocol described by Carvalho et al.^25^ The newly hatched larvae were placed in a plastic tray containing mineral water and feed composed of brewer’s yeast (35%), soybean powder (35%) and TetraMin fish feed (30%). Rearing conditions were maintained in a controlled environment (27 ± 2 °C, 65 ± 10% relative humidity, light:dark photoperiod 12:12 h). A larvicidal screening was carried out at a fixed concentration of 50 ppm for compounds **1**, **6/6′**, **9/9′**, and 4,7-dichloroquinoline. Thus, stock solutions were prepared for each compound in DMSO as a solvent at a concentration of 5 mg·mL^–1^. Next, 200 μL of each stock solution was added to beakers containing 20 larvae and 20 mL of mineral water. Equivalent systems were used for positive and negative controls. Beakers containing only 20 larvae and DMSO/water (0.1%) served as negative controls, while beakers containing only 20 larvae and Spinosad/water (0.5 ppm) served as positive controls. After 24 h, the number of live larvae was counted. For selected compounds **1**, **6′**, and **9′**, and designed compounds **2–5**, **7′**, **8′**, and **10′**, serial concentrations of 100, 50, 25, 12.5, 6.25, and 3.125 ppm were conducted to determine LC_50_ values. Then, serial concentrations were added to beakers containing 20 larvae and 20 mL of mineral water. After 24 h, the number of live larvae was counted. All experiments were performed in quintuplicate. LC_50_ values were determined by Finney’s probit regression analysis in Microsoft Excel for Windows.

### *In Silico*/*In Vitro* Toxicity Evaluation

*In silico* toxicity for 4-alkoxy/amino-7-choroquinolines (**1–5** and **6′–10′**), 4,7-dichloroquinoline, and Spinosad was predicted through the open-access electronic site pkCSM^26^ (https://biosig.lab.uq.edu.au/pkcsm/prediction). According to an adapted method reported by Fiss et al.,^19^ *in vitro* toxicity of 4-alkoxy/amino-7-choroquinolines (**1–5** and **6′–10′**) and 4,7-dichloroquinoline was evaluated on second and third instar larvae (L2–L3) of *A. salina*. *In vitro* assays on *A. salina* larvae were performed at the LPBS-UFPB. *A. salina* cysts were purchased from Bio Artemia (Grossos, Brazil). In a rectangular aquarium of 3 L capacity and using 20 W light at a height of 20 cm from the water surface, 1 g of *A. salina* cysts was placed in 1 L of saline solution (30 g·L^–1^, pH 7.5–8.5) and aerated at 27 °C for 48 h. The toxicity was evaluated at concentrations of 125, 250, and 375 μg·mL^–1^. To this end, stock solutions were prepared at a concentration of 12.5 mg·mL^–1^ for each compound, from which volumes of 50, 100, and 150 μL were captured, added to tubes, and, when necessary, completed with DMSO q.s.p. 150 μL (3%). Next, 100 μL (2%) of Tween 80 was added and completed with saline solution q.s.p. 5 mL. Then, ten healthy *A. salina* nauplii were placed inside each tube. After 24 h, the number of live nauplii was counted. Concentration series (125, 250, and 375 μg·mL^–1^) of potassium dichromate solution was used as a positive control. Two negative controls were prepared, one containing only saline solution and another containing saline solution with 3% DMSO and 2% Tween 80. All experiments were performed in triplicate. The percentage of lethality on *A. salina* (%LAS) of the samples was determined by the formula %LAS = [(number of dead nauplii in the test – number of dead nauplii in the negative control)/number of live nauplii in the negative control] × 100. Average percentages of live *A. salina* nauplii in different concentrations were calculated by using GraphPad Prism 5.0 (San Diego, California). Statistics were analyzed using the ANOVA method in a confidence interval with a confidence level of 95%. LC_50_ values were estimated using a linear regression equation in Microsoft Excel for Windows (Table S1).

## Results and Discussion

### Chemistry

Ten 4-alkoxy/amino-7-choroquinolines (**1–5** and **6′–10′**) were designed by the synthetic route shown in Scheme 1. According to methods previously described by Fiss et al.,^19^ compounds **1–10** were prepared from a Nucleophilic Aromatic Substitution (S_N_Ar) reaction of 4,7-dichloroquinoline with alcohols or amines in yields ranging from 81 to 99%. Next, according to a method previously reported by Oliveira et al.,^21^ with some adjustments, compounds **6′–10′** were obtained from a double nucleophilic substitution reaction between alcohol (**6–10**) and thionyl chloride in yields ranging from 76 to 99%.

**Scheme 1:**
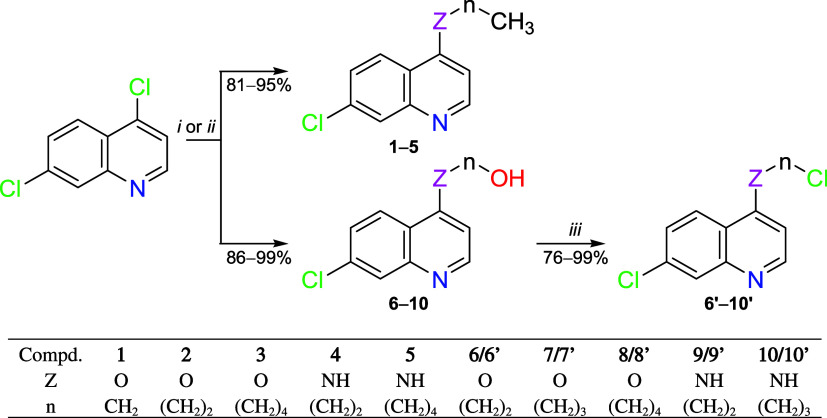
Reagents and Reactional Conditions to Obtain 4-Alkoxy/amino-7-chloroquinolines^a^

a Z = NH, (i): amine, 80 °C, 4 h (for propylamine, at 50 °C for 12 h); Z = O, (ii): alcohol, *t*-BuOK, 100 °C, 8 h (for ethanol, at 80 °C for 12 h); (iii): SOCl_2_, DCM, 25 °C, 6 h.

Full data for compounds **1**,^20^ **2–10**,^19^ and **9′–10′** ^22^ were found to be identical to the ones described previously. Compounds **6′–8′** were characterized by spectroscopic techniques of IR, ^1^H and ^13^C NMR, and HRMS analyses.

In ^1^H NMR spectra of compounds **6′–8′**, compared to the methylene hydrogens HO–CH_2_ of their precursors **6–8**, the main signal that confirms the obtaining of products is that attributed to the methylene hydrogens Cl–CH_2_, which appeared as a triplet in the range of 3.66–3.96 ppm. In addition, the absence of a signal referring to OH was observed. In ^13^C NMR spectra of compounds **6′–8′**, the methylene carbons referring to Cl–CH_2_ were observed in the range of 41.2–44.6 ppm. Finally, in relation to the IR spectra of compounds **6′–8′**, the most significant changes were the absence of a broad band related to O–H, as well as the decrease in symmetric/asymmetric stretching and in-plane/out-of-plane angular deformations related to C–O.

### *In Silico*/*In Vitro* Larvicidal Activity on *Ae. aegypti*

A larvicidal screening was carried out at a fixed concentration of 50 ppm for compounds **1**, **6/6′**, **9/9′**, and 4,7-dichloroquinoline (Table 1). Next, for selected compounds **1**, **6′**, and **9′**, and designed compounds **2–5**, **7′**, **8′**, and **10′**, LC_50_ values were determine. Furthermore, for 4-alkoxy/amino-7-choroquinolines (**1–5** and **6′–10′**), 4,7-dichloroquinoline and Spinosad, larvicidal dengue was predicted from the MolPredictX^23^ program. *In silico*/*in vitro* data are available in Table 2.

**Table 1:**
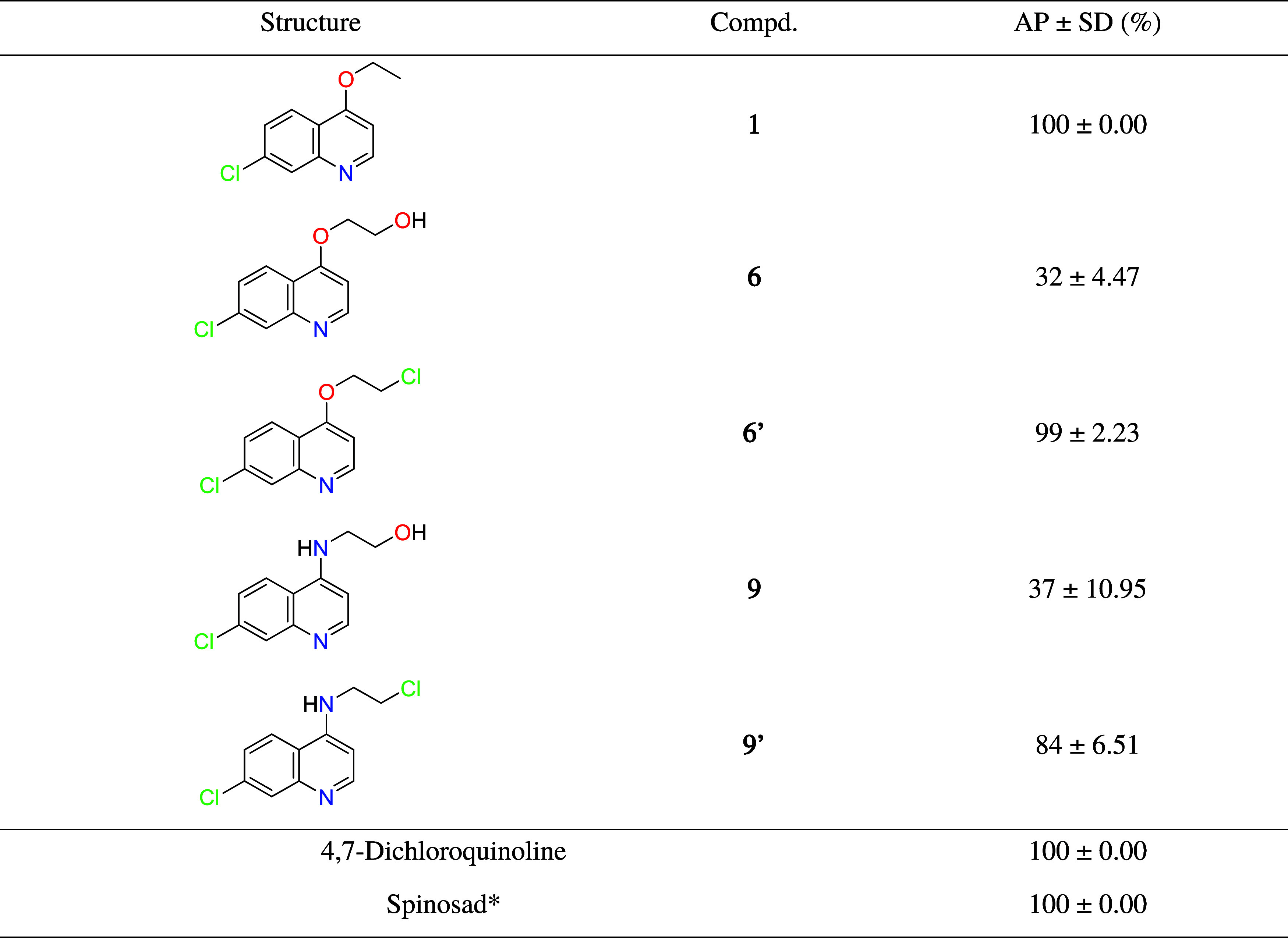
Average Percentages of Lethality on *Ae. aegypti* Larvae at 50 ppm after 24 h^a^

a AP: Average Percentage; SD: Standard Deviation. *A positive control at 0.5 ppm. All experiments were performed in quintuplicate.

**Table 2:**
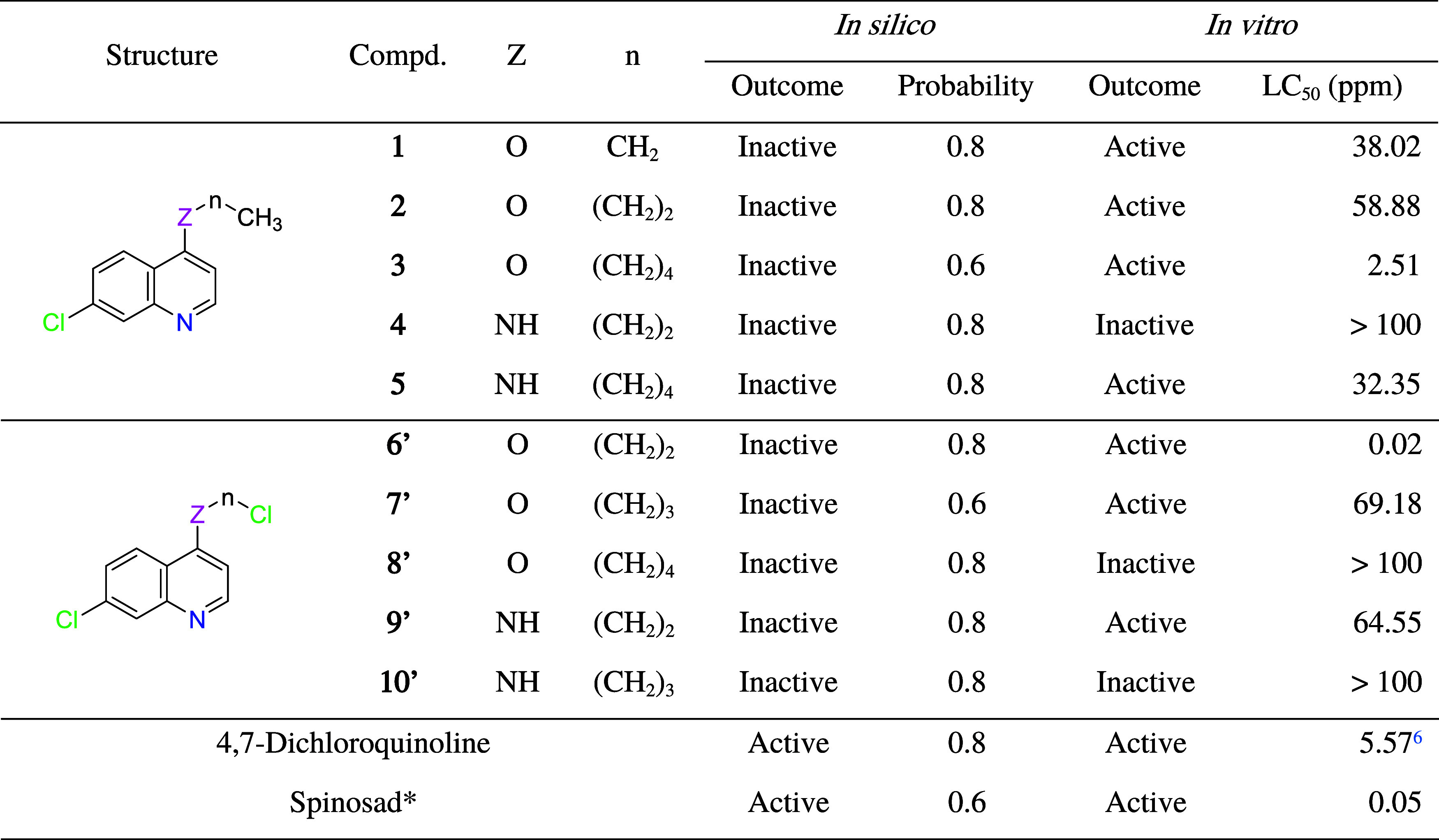
*In Silico*/*In Vitro* Larvicidal Activity on *Ae. aegypti* after 24 h^a^

a LC_50_: lethal concentration where a substance causes the death of 50% of organisms. *A positive control. Reference for LC_50_ on *Ae. aegypti* larvae after 24 h: very highly active < 1 ≤ very active < 10 ≤ active < 100.^27^

According to Table 1, initial screening revealed that the presence of hydrophilic (hydroxyl) groups in the side chain did not favor larvicidal activity. In this way, compounds **1**, **6′**, and **9′** were selected, and the series was expanded to investigate the influence of oxygen/nitrogen at the 4-position and variations in side chain length with and without a terminal chlorine atom.

About larvicidal dengue *in silico* (Table 2), 4-alkoxy/amino-7-choroquinolines (**1–5** and **6′–10′**) are predicted to be inactive, with an inactive probability of 60–80%. On the other hand, 4,7-dichloroquinoline and Spinosad are predicted to be active with an active probability of 80 and 60%, respectively. Although the MolPredictX program manages predictive models already published in indexed scientific articles and makes qualitative predictions (active or inactive) and quantitative probabilities for diverse bioactivities, theoretical studies guide but do not replace chemical intuition.

According to the World Health Organization (WHO),^27^ a potential larvicide must have an LC_50_ value of less than 100 ppm. In attention to Table 2, seven compounds were active, with LC_50_ < 100 ppm, of which compound **3** was very active, with LC_50_ < 10 ppm, and compound **6′** was very highly active, with LC_50_ < 1 ppm, greater than the Spinosad standard. Spinosad, a mixture of two isomers Spinosyn A and D, is a secondary metabolite of the aerobic fermentation of nutrients by the action of two soil bacteria of the genus *Saccharopolyspora*, which has been used as a biolarvicide against *Ae. aegypti*.^28^

For the most active compounds (**3** and **6′**), it appears that oxygen at the 4-position increased the larvicidal activity. Considering the series with a terminal chlorine atom (**6′–8′**), it seems that increasing the side chain extension decreased the larvicidal activity. Compared to a previous study for 4,7-dichloroquinoline, which showed significant larvicidal activity against *Ae. aegypti*, with LC_50_ = 5.57 ppm after 24 h,^6^ compounds **3** and **6′** demonstrated improved larvicidal effect. Furthermore, compound **6′** showed a 100% lethal concentration (LC_100_) at 7 ppm after 24 h. Therefore, the insertion of −O–pentyl and −O–ethyl–Cl moieties at the 4-position of 7-chloroquinolines appears to have enhanced the larvicidal activity. Now, it remains to evaluate the toxicity and environmental safety of these compounds.

### *In Silico*/*In Vitro* Toxicity Evaluation

Toxicity prediction for 4-alkoxy/amino-7-choroquinolines (**1–5** and **6′–10′**), 4,7-dichloroquinoline, and Spinosad was acquired from the pkCSM^26^ program. According to an adapted method reported by Fiss et al.,^19^ *in vitro* toxicity of 4-alkoxy/amino-7-choroquinolines (**1–5** and **6′–10′**) and 4,7-dichloroquinoline was evaluated on *A. salina* larvae. A saline solution, in which any nauplius died, and a saline solution containing 3% DMSO and 2% Tween 80, in which the number of dead nauplii was equal to 0.33, were used as negative controls. A potassium dichromate solution was used as a positive control in which all nauplii died. Average percentages of live *A. salina* nauplii in different concentrations are provided in Figure 2. *In silico*/*in vitro* toxicity data are summarized in Table 3.

**Figure 2:**
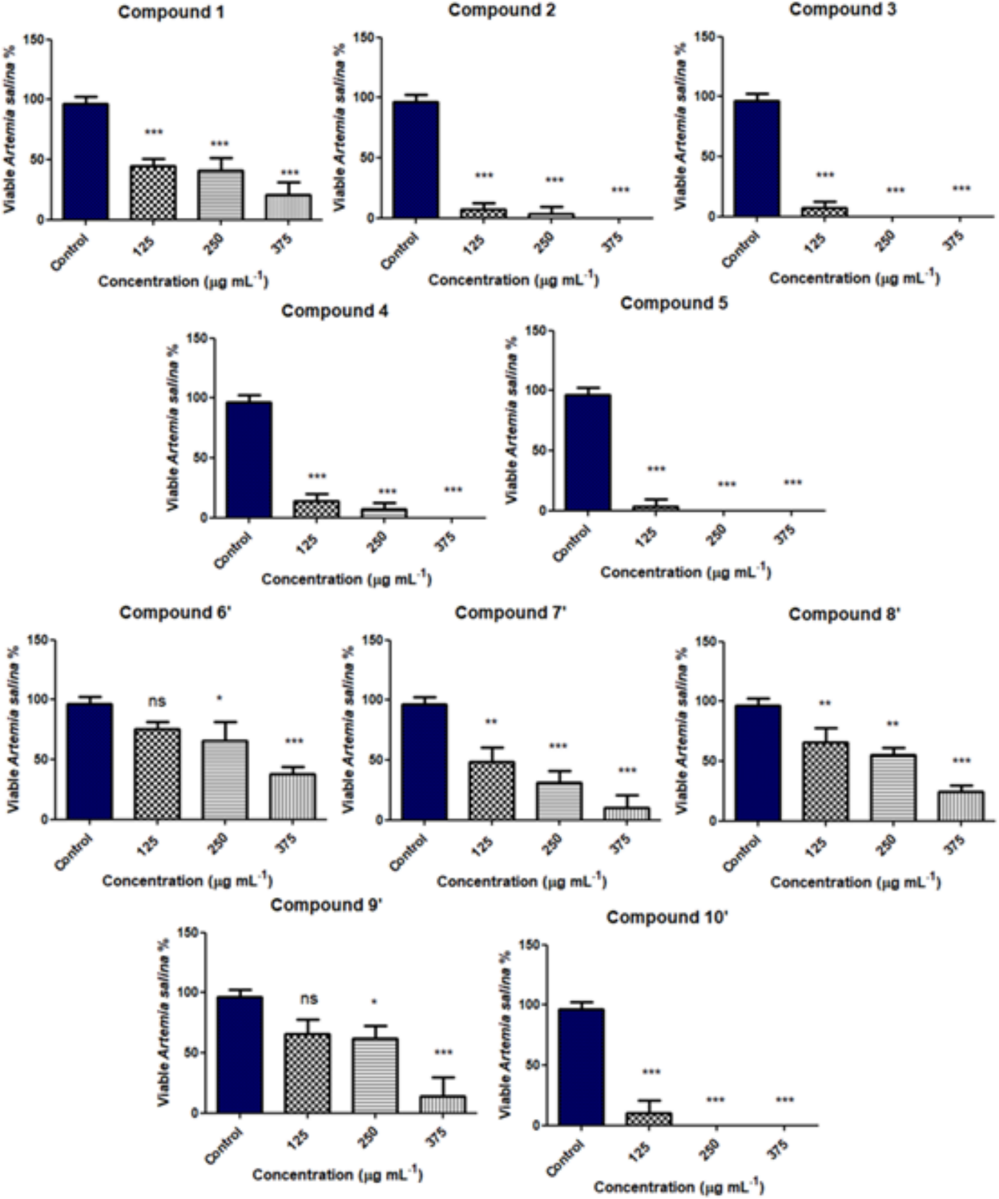
Average percentages of live *A. salina* nauplii in different concentrations of 4-alkoxy/amino-7-choroquinolines (**1–5** and **6′–10′**) after 24 h. Differences were considered statistically significant when *P* ≤ 0.05*, *P* ≤ 0.01**, *P* ≤ 0.001***, and *P* ≤ 0.0004*** using one-way analysis of variance and Bonferroni’s Multiple Comparison Test. ns: nonsignificant.

**Table 3:**
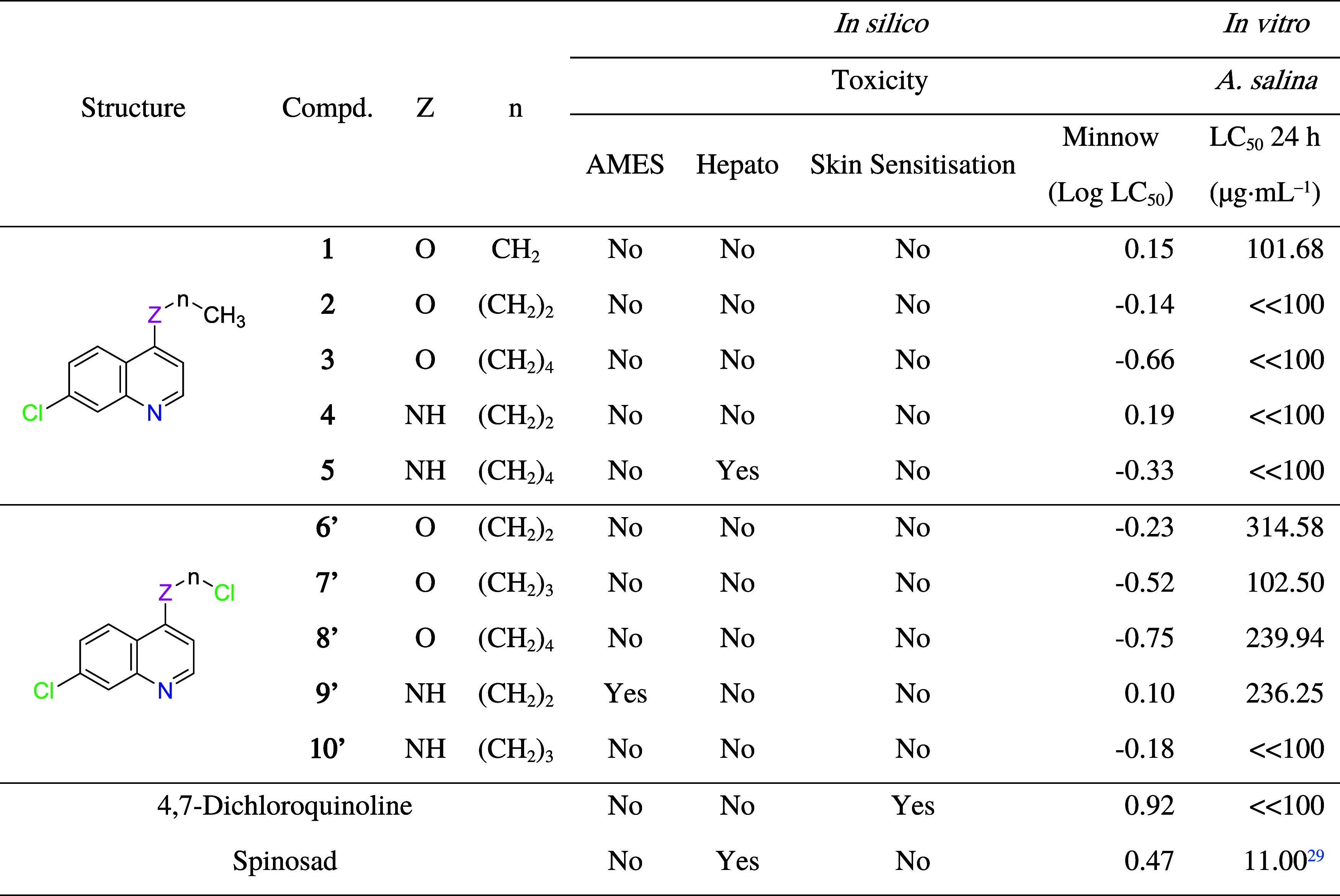
*In Silico/In Vitro* Toxicity Data^a^

a AMES: mutagenic potential using bacteria; LC_50_: lethal concentration where a substance causes the death of 50% of organisms; Minnow: LC_50_ for flathead minnows. Reference for log LC_50_ on flathead minnows: high acute toxicity < −0.3.^26^ Reference for LC_50_ on *A. salina* larvae after 24 h: highly toxic < 100 ≤ moderately toxic < 500 ≤ slightly toxic < 1000 ≤ nontoxic.^30^

According to Table 3, except for compound **9′**, compounds are potentially nonmutagenic, and except for compound **5′** and Spinosad, compounds are potentially nonhepatotoxic. On the other hand, except for 4,7-dichloroquinoline, all 4-alkoxy/amino-7-choroquinolines and Spinosad are predicted not to cause skin sensitization.

Minnow toxicity predicts LC_50_ for flathead minnows, in which compounds **3**, **5**, **7′**, and **8′** are predicted to be highly acute toxic. Regarding *in vitro* toxicity on *A. salina* larvae, compounds **3** and **5** showed high toxicity, with LC_50_ < 100 μg·mL^–1^, which coincides with the *in silico* toxicity. On the other hand, compounds **7′** and **8′** showed moderate toxicity on *A. salina* larvae, with LC_50_ values between 100 and 500 μg·mL^–1^. Surprisingly, compound **6′**, the most active against *Ae. aegypti* larvae, with LC_50_ = 0.02 ppm, was the least toxic on *A. salina* larvae, with LC_50_ = 314.58 μg·mL^–1^.

*A. salina* is commonly used as a model organism in toxicity bioassays to evaluate the ecotoxic potential of various substances, including pesticides. Although Spinosad has low toxicity to mammals and birds, and moderate toxicity to aquatic organisms, an LC_50_ value equal to 11.00 μg·mL^–1^ was reported on *A. salina* larvae after 24 h.^29^ At the recommended dosage for public control of larvicidal dengue (LC_100_ = 0.50 ppm), Spinosad presented high toxicity to the nontarget organism *Daphnia magna*.^31^ Regarding the LC_100_ value of compound **6′**, it remains safe to the nontarget organism *A. salina* even at a higher concentration of 7.00 ppm.

Considering 4-alkoxy/amino-7-choroquinolines that presented LC_50_ < 100 ppm on *Ae. aegypti* larvae and LC_50_ > 0 ppm on *A. salina* larvae, compounds **1**, **6′**, **7′**, and **9′** tend to be more toxic to *Ae. aegypti* than to the nontarget organism *A. salina* (Figure 3). Furthermore, the ecotoxicological efficacy of compounds **1**, **6′**, **7′**, and **9′** was found using the suitability index (SI), calculated by the ratio between the LC_50_ values obtained in bioassays on *A. salina* and *Ae. aegypti* larvae (Table 4).

**Figure 3:**
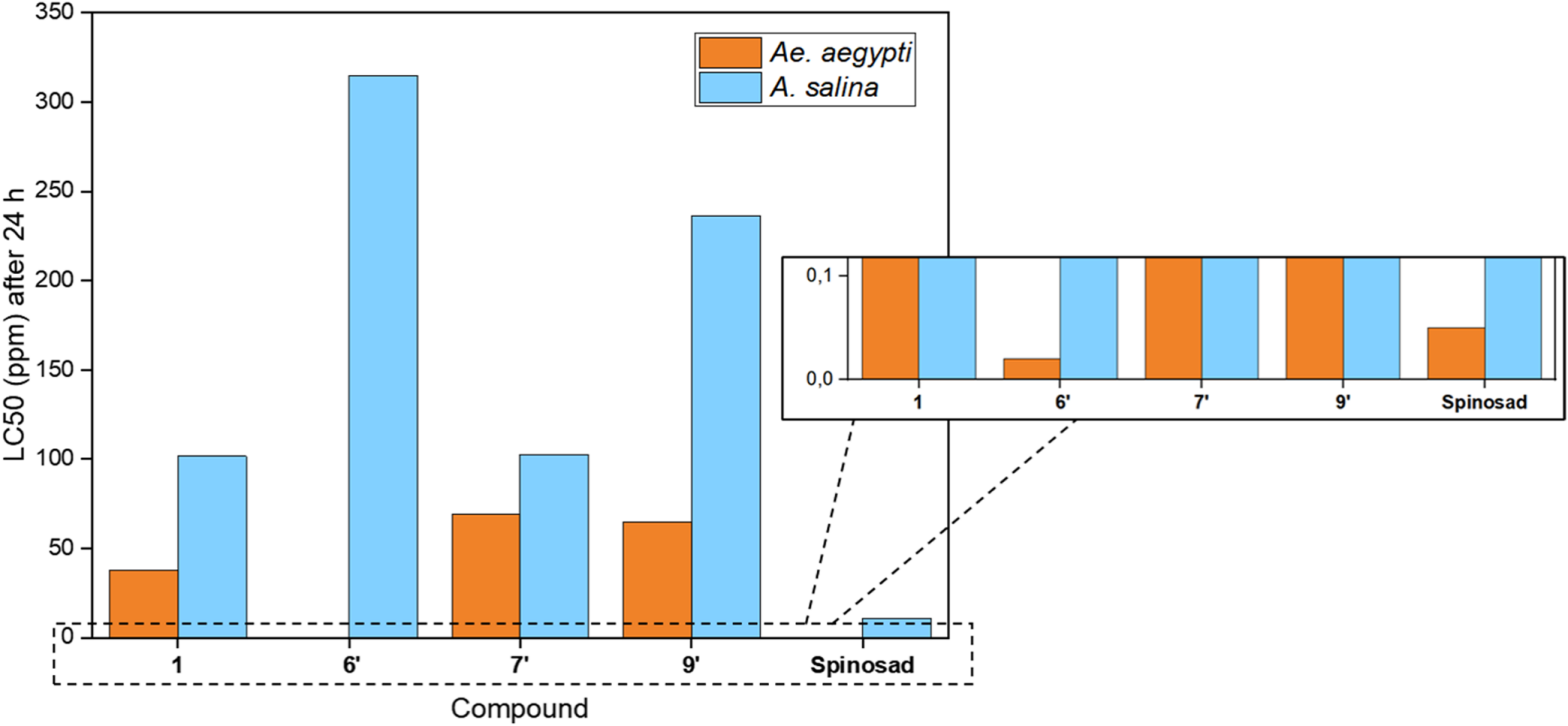
Larvicidal effect on *Ae. aegypti* and *A. salina*.

**Table 4:** SI Values on *A. salina* and *Ae. aegypti* Larvae^a^

	LC_50_ (ppm) after 24 h		
Compd.	*A. salina*	*Ae. aegypti*	SI (LC_50_ *A. salina*/LC_50_ *Ae. aegypti*)
**1**	101.68	38.02	2.67
**6′**	314.58	0.02	15 729.00
**7′**	102.50	69.18	1.48
**9′**	236.25	64.55	3.65
Spinosad	11.00 ^29^	0.05	220.00

a LC_50_: lethal concentration where a substance causes the death of 50% of organisms.

In a previous study^32^ for (−)-borneol derivatives, no correlation was found between larvicidal effects on *Ae. aegypti* and *A. salina*, suggesting different mechanisms of action on both organisms; as a result, selectivity may be conceivable, with SI values ranging from 0.02 to 8.40. According to Table 4, all compounds were more toxic against *Ae. aegypti* than on *A. salina* larvae, reaching 15 729 times for compound **6′**. This demonstrates a promising larvicidal window for 4-alkoxy/amino-7-choroquinolines. Therefore, 4-alkoxy/amino-7-choroquinolines may have more selective molecular targets, which need to be confirmed through additional studies to identify potential interactions in the mechanism of larvicidal action on *Ae. aegypti*, as well as to evaluate other nontarget organisms for a more robust ecotoxicology.

## Conclusions

Concerned about the current dengue situation in the Americas and the search for environmentally safer larvicides, three compounds were selected and seven others designed from a larvicidal screening of 4-alkoxy/amino-7-choroquinolines, of which seven compounds were active against *Ae. aegypti* larvae (LC_50_ ≤ 69.18 ppm), and five compounds were moderately toxic on *A. salina* larvae, with LC_50_ values between 101.68 and 314.58 μg·mL^–1^. Compounds **1**, **6′**, **7′**, and **9′** coincidentally showed activity against *Ae. aegypti* and moderate toxicity on *A. salina*. The best results were for 4-alkoxy-7-choroquinolines **3** and **6′**, which were very active (LC_50_ = 2.51 ppm) and very highly active (LC_50_ = 0.02 ppm) against *Ae. aegypti*, respectively, outperforming the standard larvicide Spinosad (LC_50_ = 0.05 ppm). In comparison with 4,7-dichloroquinoline, it was observed that the −O–pentyl and −O–ethyl–Cl moieties at the 4-position of 7-chloroquinolines improved the larvicidal activity. Furthermore, compound **6′** was 15 729 times more toxic to *Ae. aegypti* than to the nontarget organism *A. salina*. According to *in silico/in vitro* studies, compound **6′**, potentially nonmutagenic, nonhepatotoxic, and predicted not to cause skin sensitization, stands out as a safer larvicide and deserves further studies to investigate the molecular mechanism behind the larvicidal action.

## Supplementary Material



## Notes

Full NMR, IR, and HRMS spectra for compounds **6′–8′** are available in Figures S1–S12, and Table S1 (PDF)

## References

[1] Nicacio J. M., Gomes O. V., Carmo R. F., Nunes S. L. P., Rocha J. R. C. F., Souza C. D. F., Franca R. F. O., Khouri R., Barral-Netto M., Armstrong A. C. (2022). Heart disease and arboviruses: a systematic review
and meta-analysis. Viruses.

[2] PAHO: Pan American Health Organization https://www.paho.org/en/topics/dengue (accessed on April 10, 2025).

[3] Jemberie W., Dugassa S., Animut A. (2025). Biting hour and host seeking behavior
of *Aedes* species in urban settings, Metema District,
Northwest Ethiopia. Trop. Med. Infect. Dis..

[4] Zahid M. H., Wyk V. H., Morrison A. C., Coloma J., Lee G. O., Cevallos V., Ponce P., Eisenberg J. N. S. (2023). The
biting rate of *Aedes aegypti* and its
variability: a systematic review (1970–2022). PLoS Negl. Trop. Dis..

[5] Teich V., Arinelli R., Fahham L. (2017). *Aedes aegypti* e sociedade: o impacto econômico das arboviroses no Brasil. J. Bras. Econ. Saúde.

[6] Murugan K., Panneerselvam C., Subramaniam J., Paulpandi M., Rajaganesh R., Vasanthakumaran M., Madhavan J., Shafi S. S., Roni M., Portilla-Pulido J. S., Mendez S. C., Duque J. E., Wang L., Aziz A. T., Chandramohan B., Dinesh D., Piramanayagam S., Hwang J.-S. (2022). Synthesis of new
series of quinoline derivatives with insecticidal effects on larval
vectors of malaria and dengue diseases. Sci.
Rep..

[7] Lalithambika B., Chandrapragasam V., Mathew J., Dey P. (2023). 2-(Dec-2-enyl)-3-methyl
quinolin-4-ol–C_20_H_27_NO and 7-amino-*N*-methylphenazine-1-carboxamide–C_14_H_13_N_4_O_2_: potent bio-active compounds against
dengue vector *Aedes aegypti*. Int. J. Trop. Insect Sci..

[8] Pal P., Show S., Das S., Bhakta S., Mondal S., Roy P., Ghosh T., Nandi R. K. (2024). An efficient access to heteroaryl/aryl-annulated
pyridine derivatives and a study of their mosquito larvicidal activity
against dengue vector. Synlett.

[9] Akram M., Hameed S., Hassan A., Khan K. M. (2024). Development in the
inhibition of dengue proteases as drug targets. Curr. Med. Chem..

[10] Kaptein S. J. F., Vincetti P., Crespan E., Rivera J. I. A., Constantino G., Maga G., Neyts J., Radi M. (2018). Identification of broad-spectrum
dengue/zika virus replication inhibitors by functionalization of quinoline
and 2,6-diaminopurine scaffolds. ChemMedChem.

[11] Egan T. J., Hunter R., Kaschula C. H., Marques H. M., Misplo A., Walden J. (2000). Structure– function relationships
in aminoquinolines:
effect of amino and chloro groups on quinoline– hematin complex
formation, inhibition of β-hematin formation, and antiplasmodial
activity. J. Med. Chem..

[12] Kaschula C. H., Egan T. J., Hunter R., Basilico N., Parapini S., Taramelli D., Pasini E., Monti D. (2002). Structure–activity
relationships in 4-aminoquinoline antiplasmodials. The role of the
group at the 7-position. J. Med. Chem..

[13] Chiodi D., Ishihara Y. (2023). “Magic chloro”:
profound effects of the
chlorine atom in drug discovery. J. Med. Chem..

[14] Copetti J. P. P., Salbego P. R. S., Orlando T., Rosa J. M. L., Fiss G. F., Oliveira J. P. G., Vasconcellos M. L. A. A., Zanatta N., Bonacorso H. G., Martins M. A. P. (2020). Substituent effects
on the crystallization mechanisms of 7-chloro-4-substituted-quinolines. CrystEngComm.

[15] Olmedo D. A., Vásquez Y., Morán J. A., De Léon E. G., Caballero-George C., Solís P. N. (2024). Understanding the *Artemia salina* (brine shrimp) test: pharmacological
significance and global impact. Comb. Chem.
High Throughput Screen..

[16] Nguyen H. H., Nguyen C. T., Ngo H. G., Nguyen G. T. T., Thuy P. T., Setzer W. N., Kuo P.-C., Bui H. M. (2023). Potential for *Aedes aegypti* larval control and environmental friendliness
of the compounds containing 2-methyl-3,4-dihydroquinazolin-4-one heterocycle. ACS Omega.

[17] Huang M.-F. N., Luis J. A. S., Silva A. P., Rocha J. C., Lima T. K. S., Scotti M. T., Scotti L., Oliveira R. F., Souza H. D. S., Athayde-Filho P. F., Barbosa-Filho J. M. (2021). Synthesis, *in silico* study and antileishmanial evaluation of new selenides
derived from 7-chloro-quinoline and *N*-phenylacetamides. J. Braz. Chem. Soc..

[18] Costa D. P., Sousa A. P., Cardoso L. L., Vanderley S. E. R., Almeida F. S., Keesen T. S. L., Brito T. A. M., Silva M. S., Athayde-Filho P. F., Fiss G. F. (2025). Novel 2-acetanilide
2-arylquinoline-4-carboxylates
as antileishmanial agents: from prediction to in vitro activity/toxicity. Curr. Org. Chem..

[19] Fiss G. F., Silva E. P., Madruga M. F. S., Sousa A. P., Souza H. D. S., Castor R. B., Nascimento M. H., Lira K. G., Athayde-Filho P. F. (2025). Athayde-Filho,
P.F. Side chain effects on the lipophilicity–antimicrobial–toxicity
correlation of greener 4-alkoxy/amino-7-chloroquinolines. Curr. Med. Chem..

[20] Heindel N. D., Fine S. A. (1969). Acid catalyzed alcoholysis of 4,7-dichloroquinoline. J. Heterocycl. Chem..

[21] Oliveira J. P. G., Caleffi G. S., Silva E. P., Coelho M. C., Castro A. C., Mendes R. K. S., Olegário T. R., Lima-Junior C. G., Vasconcellos M. L. A. A., Souza J. L. C., Souza S. M., Militão G. C. G., Vaz B. G., Ramalho R. R. F. (2021). Morita-Baylis-Hillman
reaction with 7-chloroquinoline derivatives–new compounds with
potential anticancer activity. J. Braz. Chem.
Soc..

[22] Njogu P. M., Gut J., Rosenthal P. J., Chibale K. (2013). Design, synthesis, and antiplasmodial
activity of hybrid compounds based on (2*R*,3*S*)-*N*-benzoyl-3-phenylisoserine. ACS Med. Chem. Lett..

[23] Tullius
Scotti M., Herrera-Acevedo C., Barros de Menezes R. P., Martin H.-J., Muratov E. N., Italo de Souza Silva Á., Faustino Albuquerque E., Ferreira Calado L., Coy-Barrera E., Scotti L. (2022). MolPredictX: online biological activity
predictions by machine learning models. Mol.
Inf..

[24] Almeida
e Sá F. H., Silva A. R. N., Oliveira T. J. S., Guimarães A. L., Azevedo F. R., Santos M. B., Pinto A. T. M., Virginio J. F., Alencar-Filho E. B. (2023). A chalcone identified by *in silico* and *in vitro* assays possesses high larvicidal activity
against *Aedes aegypti*. Acta Trop..

[25] Carvalho D. O., Nimmo D., Naish N., McKemey A. R., Gray P., Wilke A. B. B., Marrelli M. T., Virginio J. F., Alphey L., Capurro M. L. (2014). Mass production
of genetically modified *Aedes aegypti* for field releases in Brazil. J. Vis. Exp..

[26] Pires D. E. V., Blundell T. L., Ascher D. B. (2015). pkCSM:
Predicting small-molecule
pharmacokinetic and toxicity properties using graph-based signatures. J. Med. Chem..

[27] WHO: World Health Organization . Guidelines for Laboratory and Field Testing of Mosquito Larvicides. https://www.who.int/publications/i/item/WHO-CDS-WHOPES-GCDPP-2005.13 (accessed on April 10, 2025).

[28] Santos V. S. V., Pereira B. B. (2020). Properties, toxicity and current applications of the
biolarvicide spinosad. J. Toxicol. Environ.
Health, Part B.

[29] Mogas, A. R. M. Y. A combinação simultânea de peixes larvívoros com pesticidas como uma estratégia de controlo de vetores da malária-Um estudo experimental com Poecilia reticulata e três pesticidas 2016. https://repositorio-aberto.up.pt/handle/10216/90735?locale=en (accessed on April 10, 2025).

[30] Nguta J. M., Mbaria J. M., Gakuya D. W., Gathumbi P. K., Kabasa J. D., Kiama S. G. (2011). Biological screening of Kenyan medicinal plants using *Artemia salina* L. (Artemiidae). Pharmacologyonline.

[31] Santos V. S. V., Silva C. E., Oliveira C. M., Morais C. R., Limongi J. E., Pereira B. B. (2019). Evaluation of toxicity and environmental safety in
use of spinosad to rationalize control strategies against *Aedes aegypti*. Chemosphere.

[32] Nunes R. K. V., Martins U. N., Brito T. B., Nepel A., Costa E. V., Barison A., Santos R. L. C., Cavalcanti S. C. H. (2018). Evaluation
of (−)-borneol derivatives against the Zika vector, *Aedes aegypti* and a non-target species, *Artemia* sp. Environ. Sci. Pollut. Res. Int..

