# Supplementary material for: Effects of 4‑Alkoxy/Amino-7-Chloroquinolines on Aedes aegypti and Artemia salina Larvae: The Search for Safer Larvicides to Combat Epidemiological Dengue in the Americas
Source: ACS Omega. 2025 Aug 15;10(33):37906–15. doi: 10.1021/acsomega.5c05060 (PMC12391930; doi:10.1021/acsomega.5c05060)
Supplement: Supplementary file 1 [file ao5c05060_si_001.pdf]

Effects of 4-Alkoxy/Amino-7-Chloroquinolines on *Aedes aegypti* and *Artemia salina* larvae: the Search for Safer Larvicides to Combat Epidemiological Dengue in the Americas

*Everton P. Silva, Agenor P. Luz-Filho, Abraão P. Sousa, Edilson B. Alencar-Filho, Vanessa C. Santos, Luana B. R. Silva, Priscila S. V. Lima, Helivaldo D. S. Souza, Petrônio F. Athayde-Filho, Gabriela F. Fiss**

Gabriela F. Fiss – *Department of Chemistry, Universidade Federal da Paraíba (UFPB), João Pessoa 58051-900, Brazil;* Emails: gffiss@gmail.com, gff@academico.ufpb.br

Everton P. Silva – *Department of Chemistry, Universidade Federal da Paraíba (UFPB), João Pessoa 58051-900, Brazil*

Agenor P. Luz-Filho – *Department of Chemistry, Universidade Federal da Paraíba (UFPB), João Pessoa 58051-900, Brazil*

Abraão P. Sousa – *Department of Chemistry, Universidade Federal da Paraíba (UFPB), João Pessoa 58051-900, Brazil*

Edilson B. Alencar-Filho – *Department of Pharmaceutical Sciences, Universidade Federal do Vale do São Francisco (UNIVASF), Petrolina 56304-917, Brazil*

Vanessa C. Santos – *Department of Pharmaceutical Sciences, Universidade Federal do Vale do São Francisco (UNIVASF), Petrolina 56304-917, Brazil*

Luana B. R. Silva – *Department of Pharmaceutical Sciences, Universidade Federal do Vale do São Francisco (UNIVASF), Petrolina 56304-917, Brazil*

Priscila S. V. Lima – *Department of Chemistry, Universidade Federal de Santa Maria (UFSM), Santa Maria 97105-900, Brazil*

Helivaldo D. S. Souza – *Department of Chemistry, Universidade Federal da Paraíba (UFPB), João Pessoa 58051-900, Brazil*

Petrônio F. Athayde-Filho – *Department of Chemistry, Universidade Federal da Paraíba (UFPB), João Pessoa 58051-900, Brazil*

Supporting Information. Full NMR, IR and HRMS spectra for compounds **6'–8'** are available in Figures S1–S12, and Table S1.

Figure S1. ^{1}H NMR spectrum (500 MHz, $CDCl_3$) of compound **6'**.

Figure S2. ^{13}C NMR spectrum (126 MHz, $CDCl_3$) of compound **6'**.

Figure S3. IR spectrum (KBr) of compound **6'**.

Figure S4. HRMS spectrum (ESI) of compound **6'**.

Figure S5. ^{1}H NMR spectrum (500 MHz, $CDCl_3$) of compound **7'**.

Figure S6. ^{13}C NMR spectrum (126 MHz, $CDCl_3$) of compound **7'**.

Figure S7. IR spectrum (KBr) of compound **7'**.

Figure S8. HRMS spectrum (ESI) of compound **7'**.

Figure S9. ^{1}H NMR spectrum (500 MHz, $CDCl_3$) of compound **8'**.

Figure S10. ^{13}C NMR spectrum (126 MHz, $CDCl_3$) of compound **8'**.

Figure S11. IR spectrum (KBr) of compound **8'**.

Figure S12. HRMS spectrum (ESI) of compound **8'**.

Table S1. Estimated 50% lethal concentration (LC_{50}) of 4-alkoxy/amino-7-choroquinolines (**1–5** and **6'–10'**) and 4,7-dichloroquinoline on *Artemia salina* larvae after 24 h using linear regression equation.

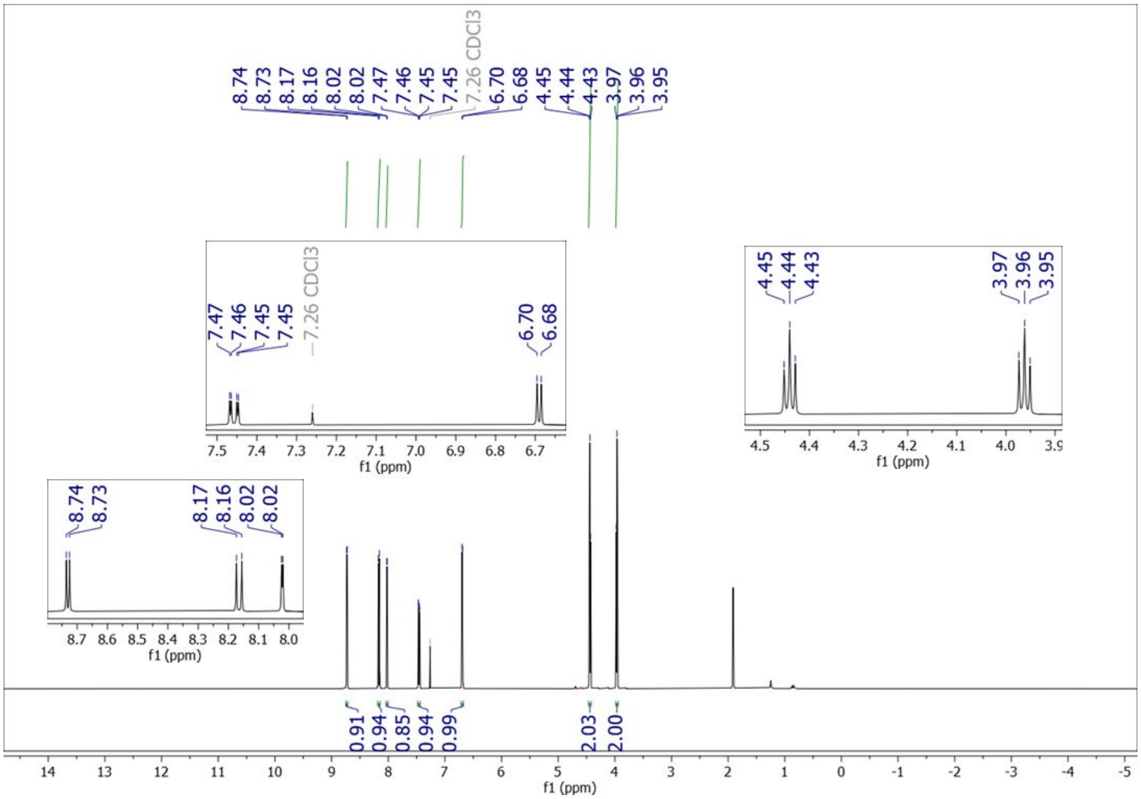

Figure S1. ^{1}H NMR spectrum (500 MHz, $CDCl_3$) of compound **6'**.

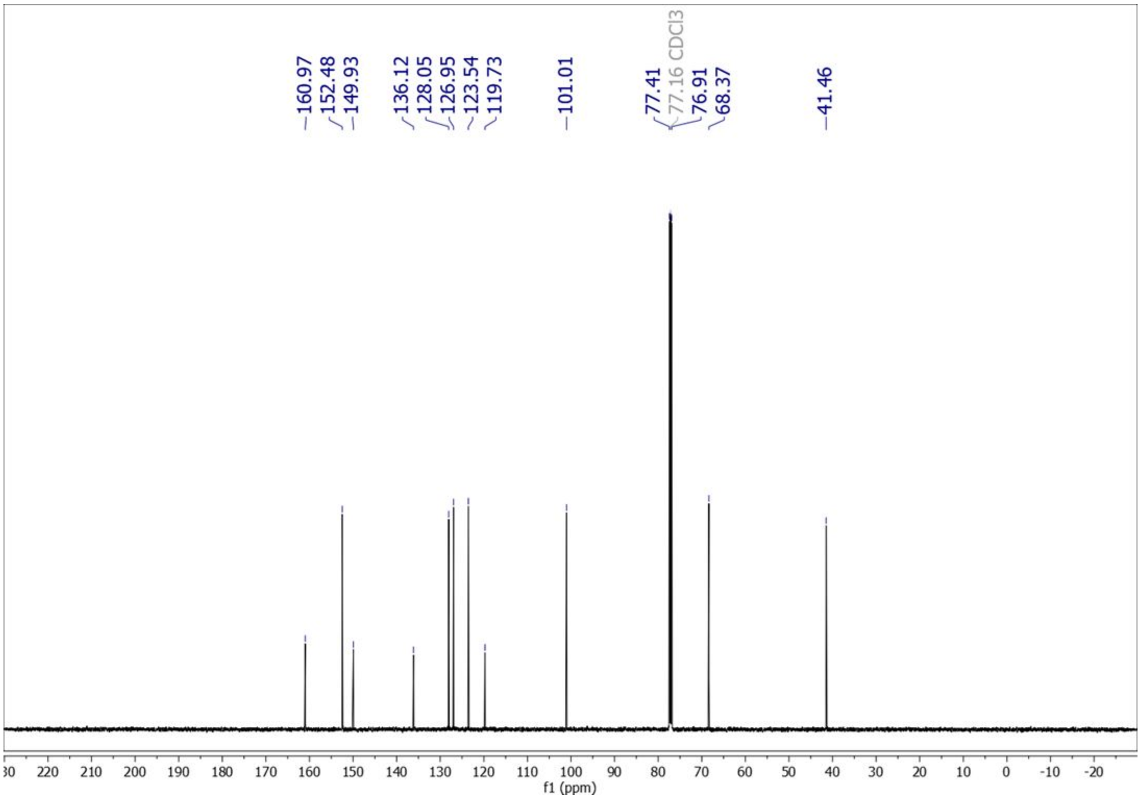

Figure S2. ^{13}C NMR spectrum (126 MHz, $CDCl_3$) of compound **6'**.

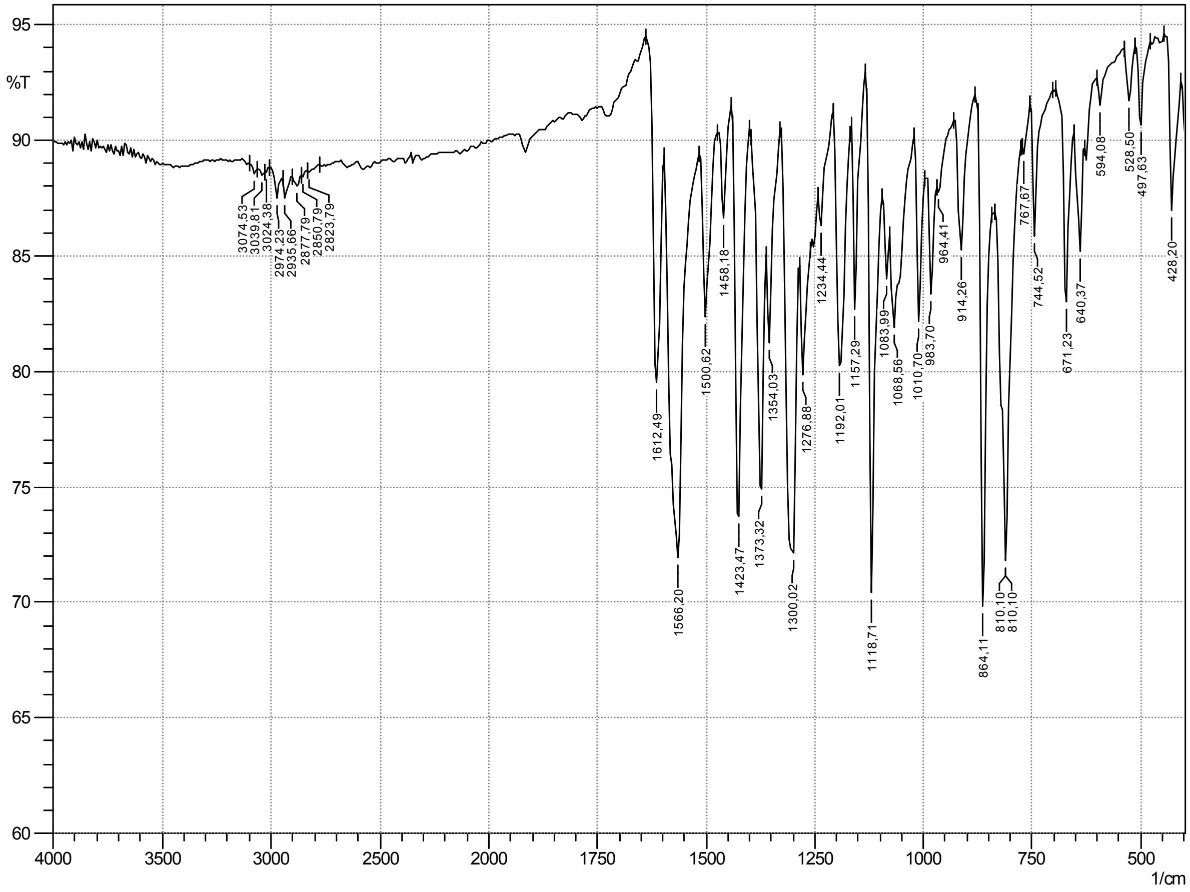

Figure S3. IR spectrum (KBr) of compound **6'**.

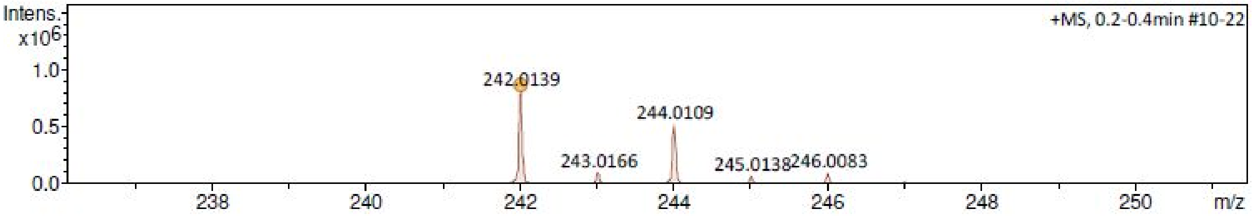

Meas. m/z	#	Ion Formula	m/z	err [ppm]	mSigma	# mSigma	Score	rdb	e⁻ Conf	N-Rule
242.0139	1	C11H10Cl2NO	242.0134	-2.2	2.2	1	100.00	6.5	even	ok

Figure S4. HRMS spectrum (ESI) of compound **6'**.

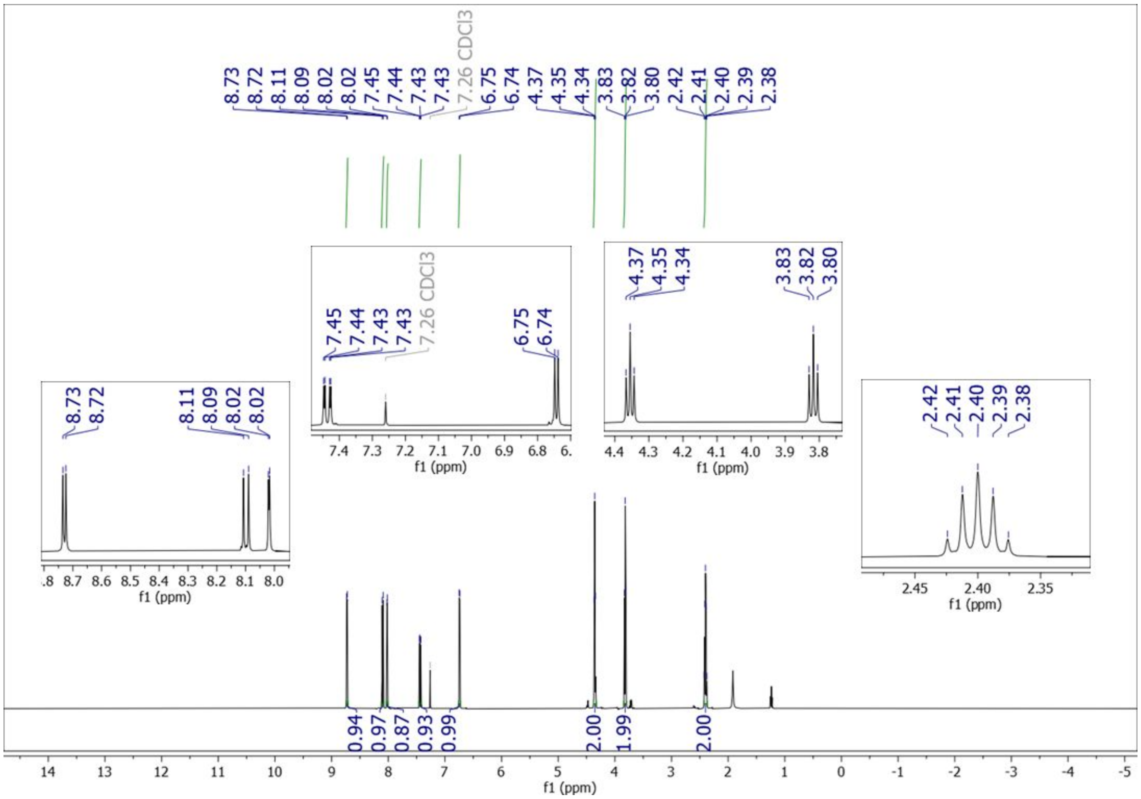

Figure S5. ^{1}H NMR spectrum (500 MHz, $CDCl_3$) of compound **7'**.

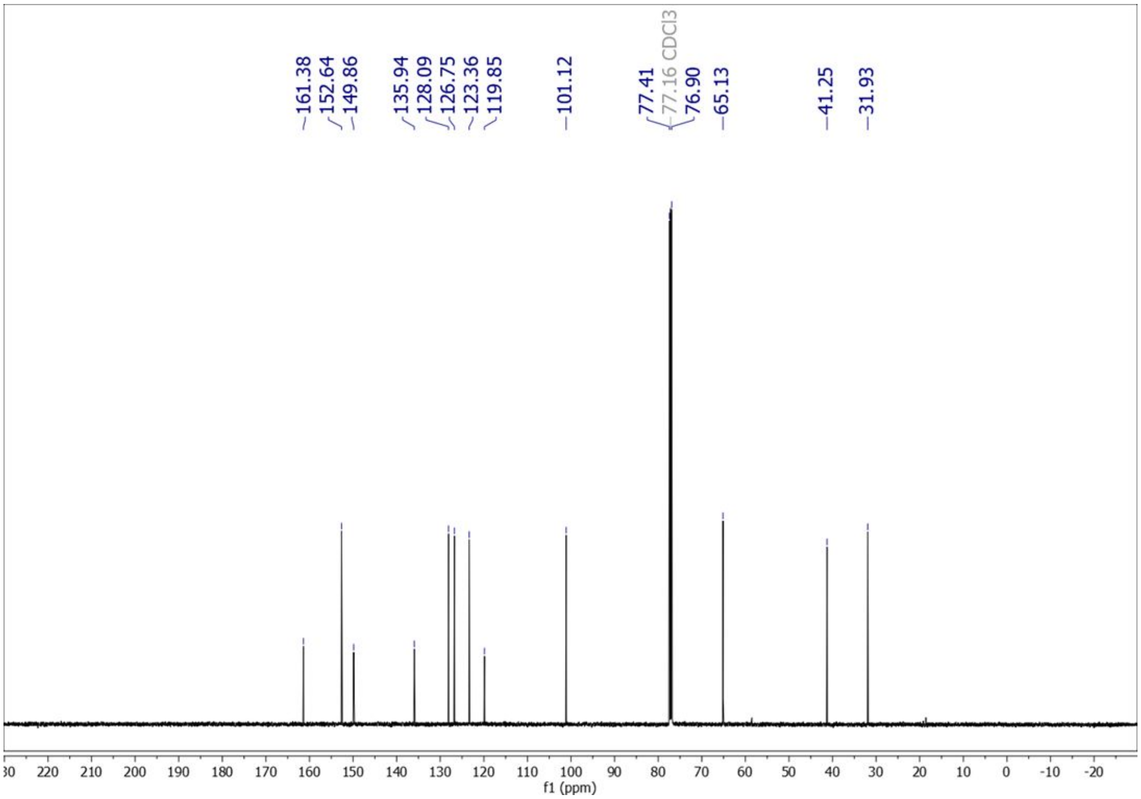

Figure S6. ^{13}C NMR spectrum (126 MHz, $CDCl_3$) of compound **7’**.

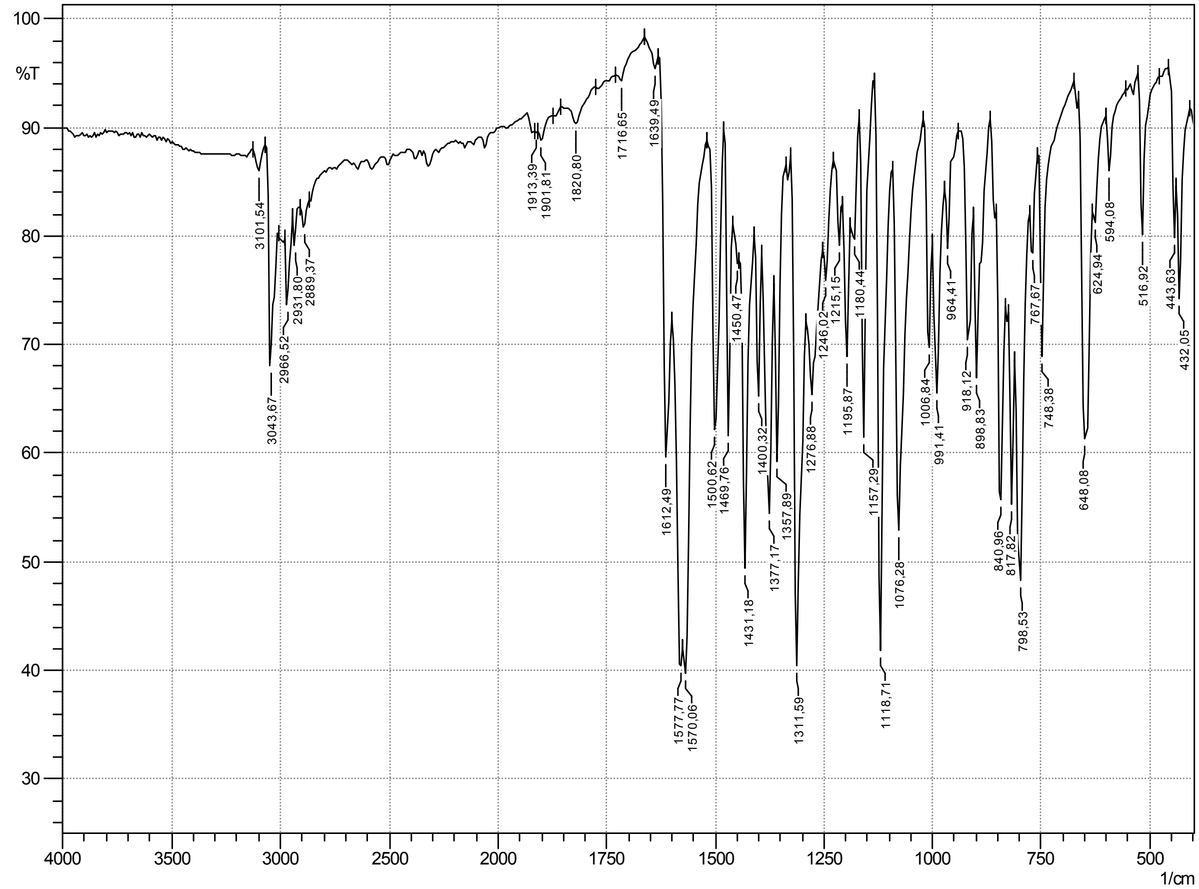

Figure S7. IR spectrum (KBr) of compound **7’**.

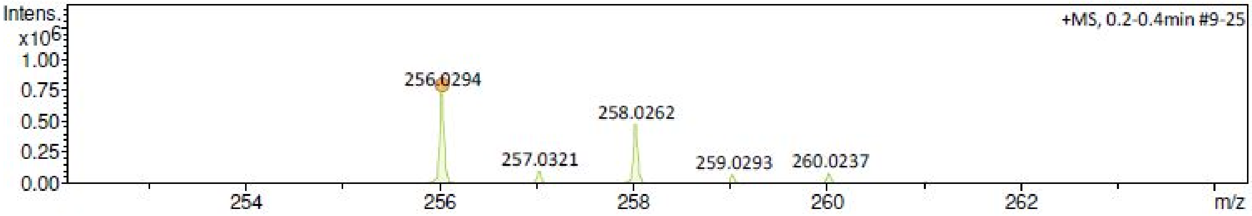

Meas. m/z	#	Ion Formula	m/z	err [ppm]	mSigma	# mSigma	Score	rdb	e⁻ Conf	N-Rule
256.0294	1	C12H12Cl2NO	256.0290	-1.4	2.3	1	100.00	6.5	even	ok

Figure S8. HRMS spectrum (ESI) of compound **7’**.

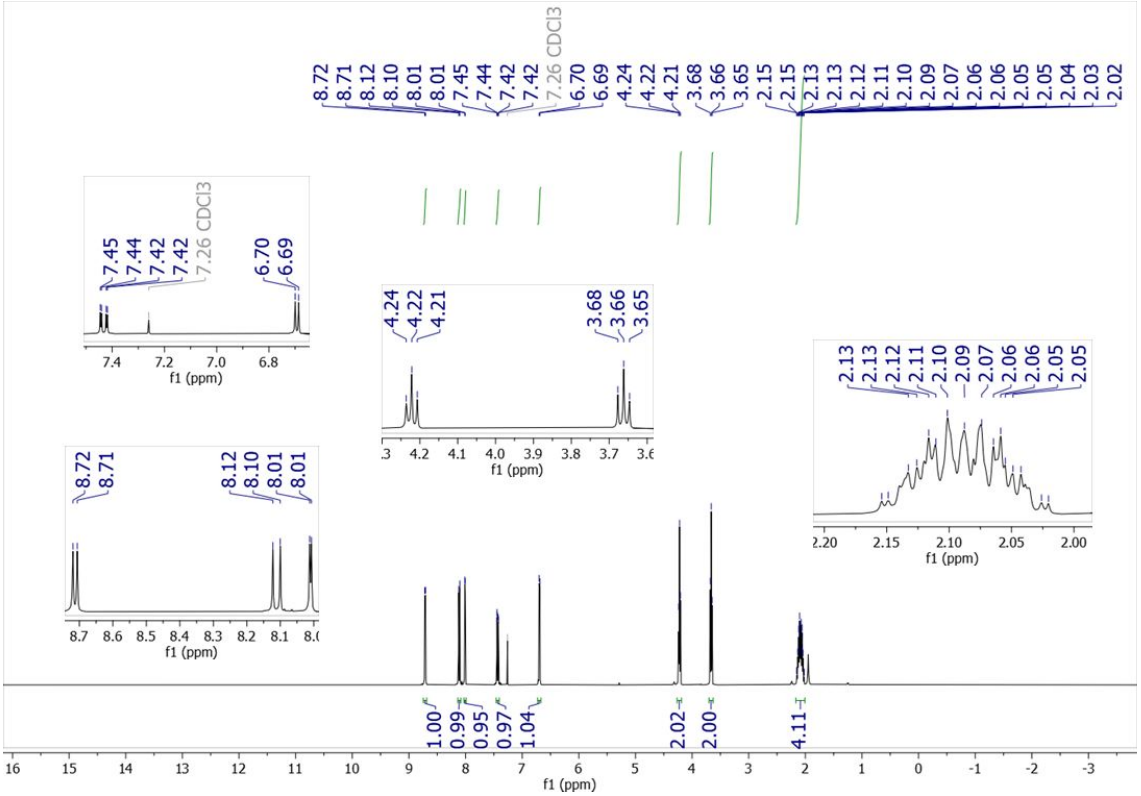

Figure S9. ^{1}H NMR spectrum (500 MHz, $CDCl_3$) of compound **8'**.

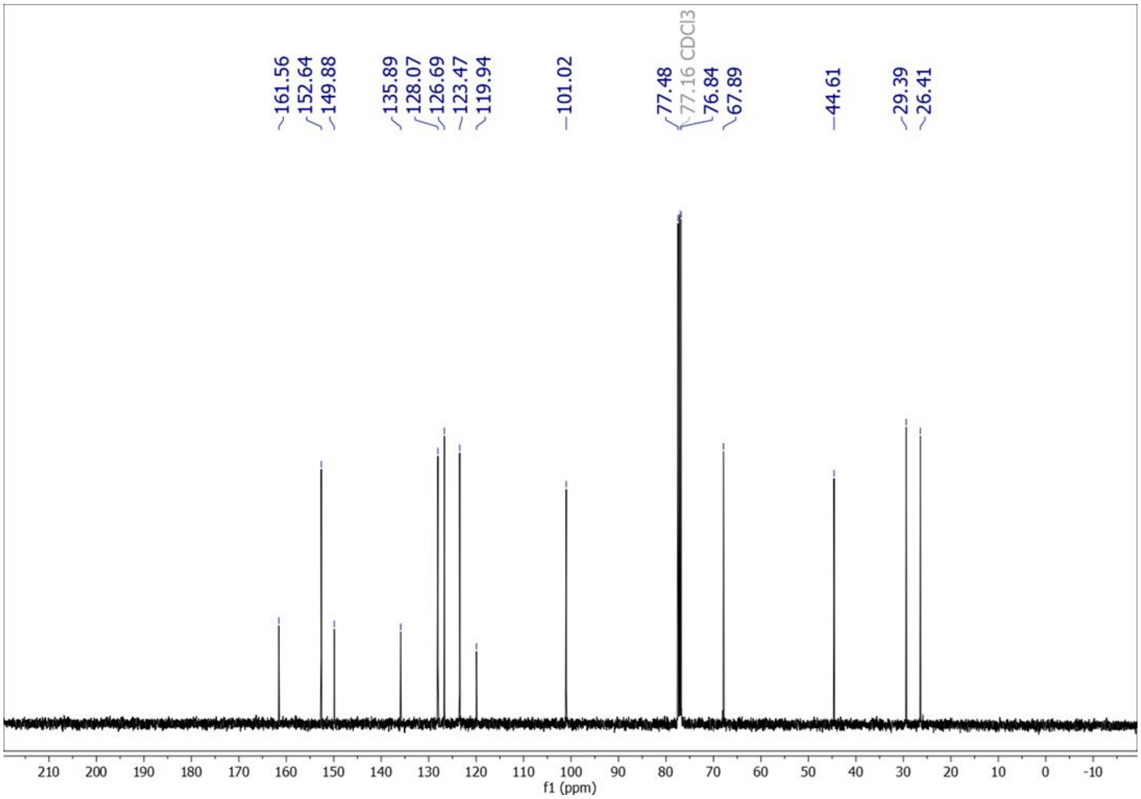

Figure S10. ^{13}C NMR spectrum (126 MHz, $CDCl_3$) of compound **8'**.

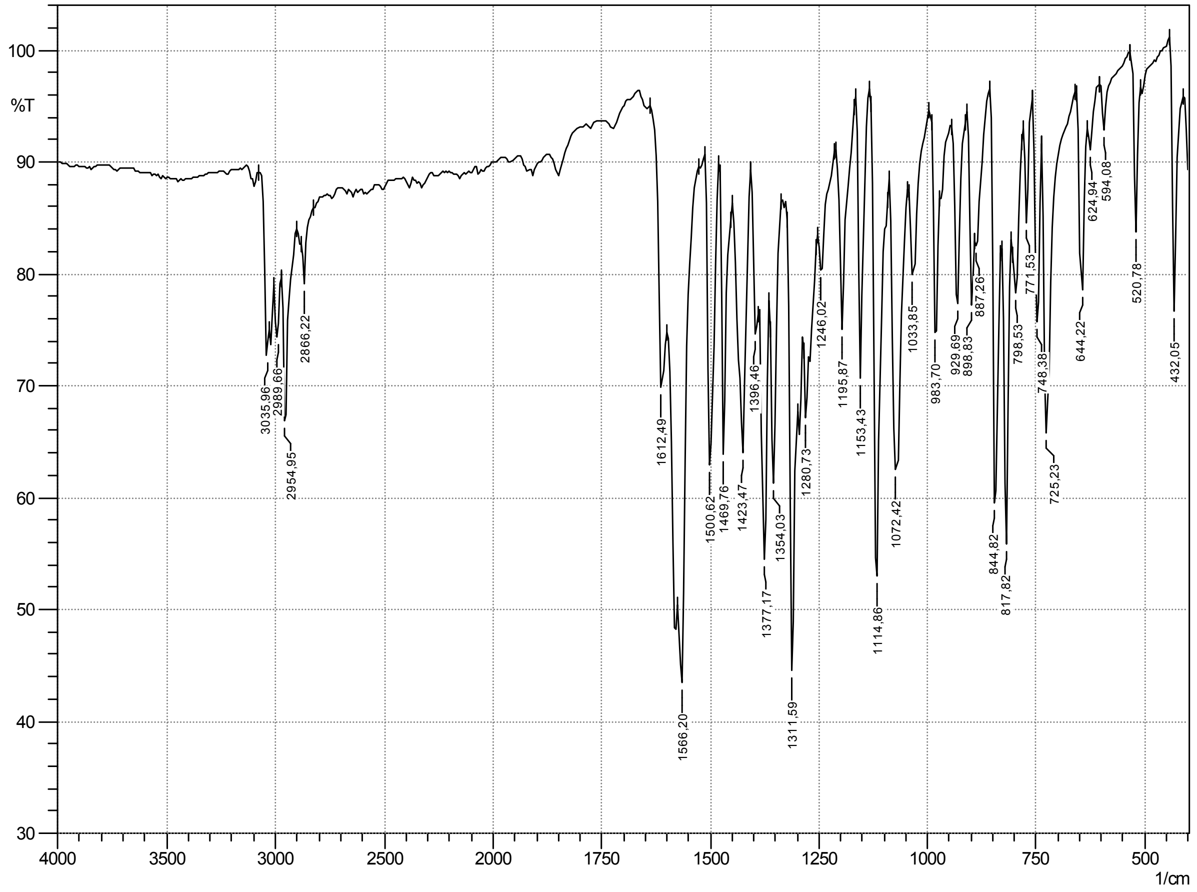

Figure S11. IR spectrum (KBr) of compound **8'**.

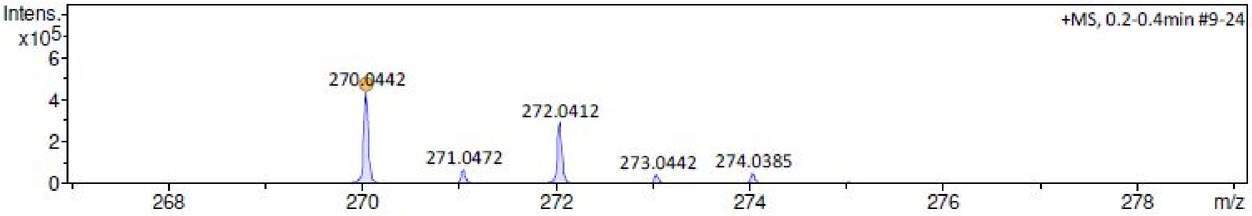

Meas. m/z	#	Ion Formula	m/z	err [ppm]	mSigma	# mSigma	Score	rdb	e⁻ Conf	N-Rule
270.0442	1	C13H14Cl2NO	270.0447	1.8	5.5	1	100.00	6.5	even	ok

Figure S12. HRMS spectrum (ESI) of compound **8'**.

Table S1. Estimated 50% lethal concentration (LC_{50}) of 4-alkoxy/amino-7-choroquinolines (**1–5** and **6’–10’**) and 4,7-dichloroquinoline on *Artemia salina* larvae after 24 h using linear regression equation.

Compd.	Graphical
1	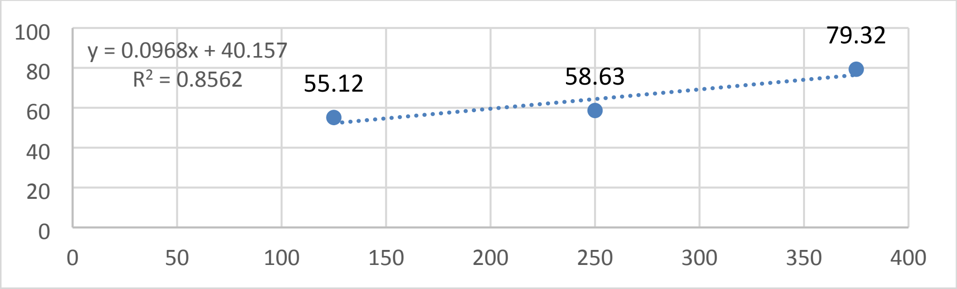
2	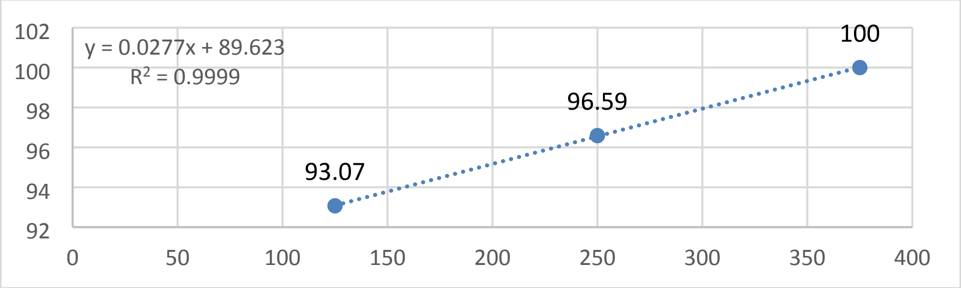
3	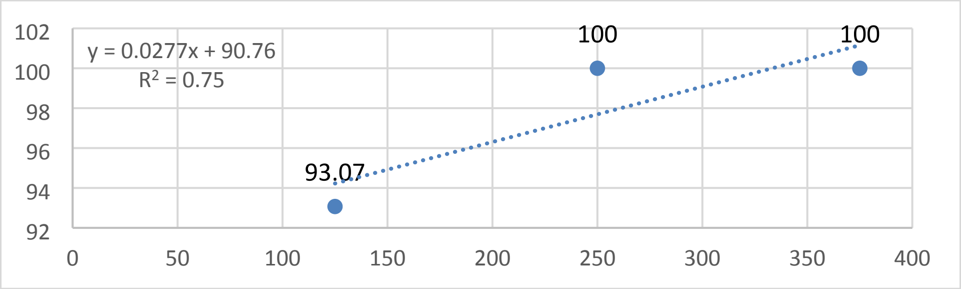
4	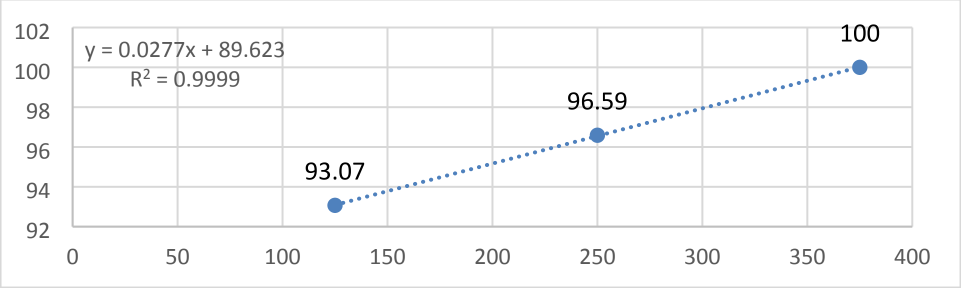

5

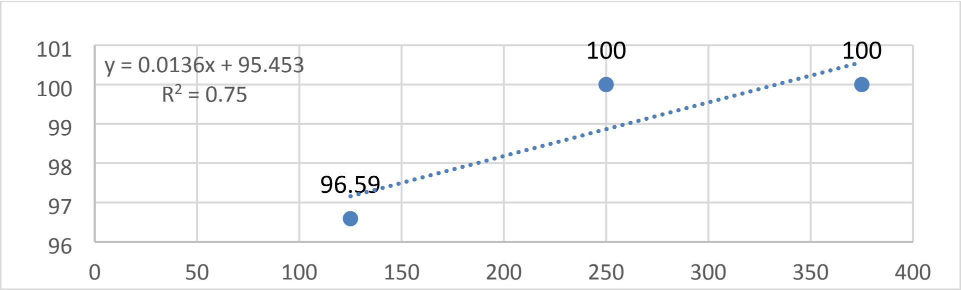
y = 0.0136x + 95.453
R² = 0.75
100
100
96.59
101
100
99
98
97
96
0
50
100
150
200
250
300
350
400

6’

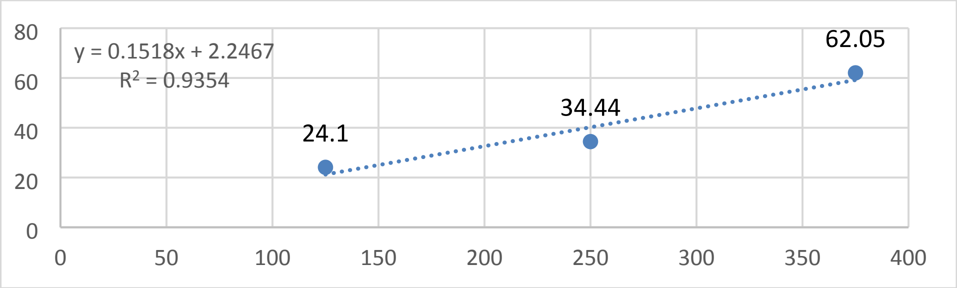
y = 0.1518x + 2.2467
R² = 0.9354
62.05
34.44
24.1
80
60
40
20
0
0
50
100
150
200
250
300
350
400

7’

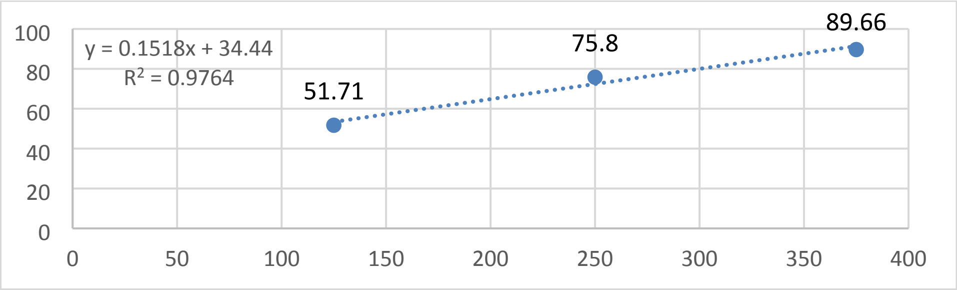
y = 0.1518x + 34.44
R² = 0.9764
89.66
75.8
51.71
100
80
60
40
20
0
0
50
100
150
200
250
300
350
400

8’

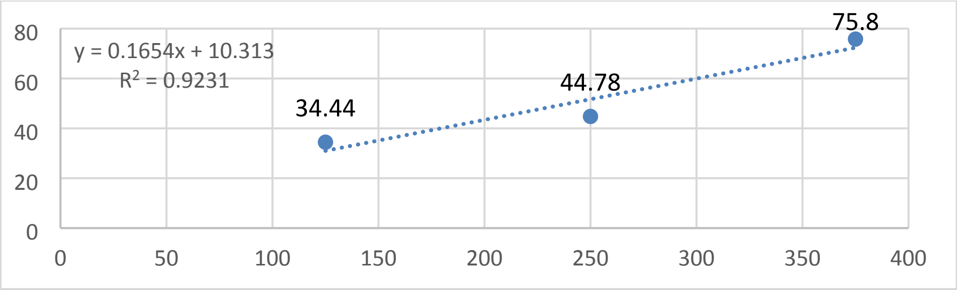
y = 0.1654x + 10.313
R² = 0.9231
75.8
44.78
34.44
80
60
40
20
0
0
50
100
150
200
250
300
350
400

9’

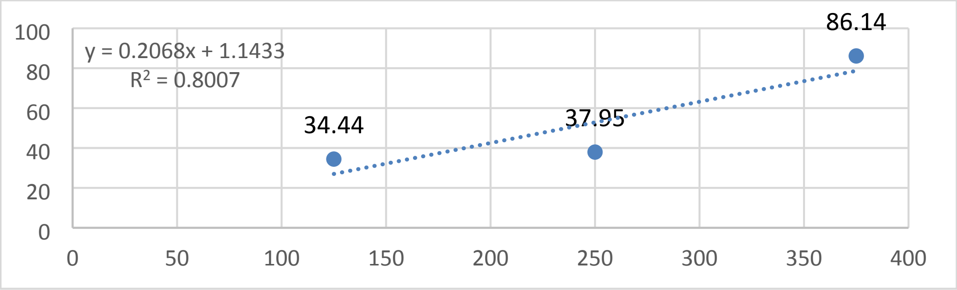
y = 0.2068x + 1.1433
R² = 0.8007
86.14
37.95
34.44
100
80
60
40
20
0
0
50
100
150
200
250
300
350
400

10’	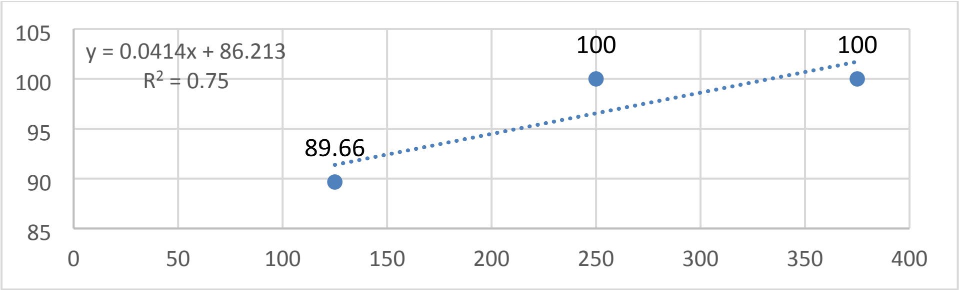
4,7-Dichloroquinoline	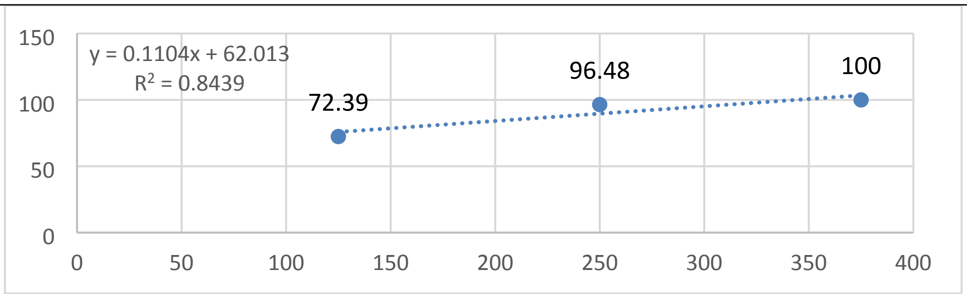
